# Supplementary material for: Digenic inheritance involving a muscle-specific protein kinase and the giant titin protein causes a skeletal muscle myopathy
Source: Nat Genet. 2024 Mar 1;56(3):395–407. doi: 10.1038/s41588-023-01651-0 (PMC10937387; doi:10.1038/s41588-023-01651-0)
Supplement: Supplementary file 1 — Supplementary Note. [file 41588_2023_1651_MOESM1_ESM.pdf]

nature genetics

Article https://doi.org/10.1038/s41588-023-01651-0

Digenic inheritance involving a muscle-specific protein kinase and the giant titin protein causes a skeletal muscle myopathy

In the format provided by the authors and unedited

Table of Contents

Ethics and consents 2
Next generation sequencing 2
Segregation analysis 4
X-inactivation assay 4
Statistical analysis of variant frequencies 5
Statistical modelling of familial segregation 7
References 32

Ethics and consents

All clinical information and biological material used in this collaborative study was collected after obtaining written informed consent from the patients or their legal guardians. Each sequencing study was approved by their relevant Health Research Authorities, as follows:

Family A: Health Research Authority, NRES Committee East of England – Hatfield (REC 06/Q0406/33). Families B, W and X: Consent approved by the French legislation (Comité de Protection des Personnes Est IV DC-2012-1693). DNA storage and usage was IRB-approved: Comités de Protection des Personnes (CPP-Est DC-2012-1693). Family C: Ethics Committee of the National Center of Neurology and Psychiatry, Japan (A2011-081). Families D, R, V, Y and Z: NRES Committee North East – Newcastle & North Tyneside 1 (REC 08/H0906/28+5). Family E: EC approval (Number S52853) by Ethical Committee Research UZ / KU Leuven. Families H and U: by the Medical Review Ethics Committee, Region Arnhem–Nijmegen, Number 2011/188. Family L: NIH, National Institute of Neurological Disorders and Stroke (NINDS), Institutional Review Board (Protocol 12-N-0095). Family M: Ethics committee of the Helsingin ja Uudenmaan sairaanhoitopiiri (HUS, statement number 195/13/03/00/11). Family N: University of Pretoria Faculty of Health Sciences Research Ethics Committee Ref 296/2019. Families O and P: Boston Children's Hospital Institutional Review Board (Protocol #03-08-128R). Family Q: Rare Genomes Project (Protocol #: 2016P001422) study, approved by the IRB at Massachusetts General Brigham. Family S: New Zealand Health and Disability Ethics Committee - approval number 20/NTB/139. Families F, G, J, K and T: tested through their respective national diagnostic health services.

Next generation sequencing

Patient's genomic DNA extracted from peripheral blood was subjected to next generation sequencing (NGS), either exome or panel sequencing. The genetic data was analysed using standard

filtering criteria for rare diseases including minor allele frequency of <1% in the gnomAD control population (https://gnomad.broadinstitute.org/), high to moderate effect on protein structure (i.e. nonsense, splice sites, frameshift, non-synonymous and in-frame variants) and an optional neuromuscular disease gene list. The specific sequencing methods used by each collaborating center are listed below.

Families A and Y were whole exome sequenced as part of the NeurOmics project at deCODE genetics, Iceland, using Illumina Nextera Rapid Capture exome kit (37 Mb). Families F and K were sequenced through the Congenital Myopathy Panel at Guy's Hospital, London, UK. Families B, W and X were whole exome sequenced as part of the Myocapture project as described in ref. 1. Families D, E, R, V and Z were whole exome sequenced as part of the MYO-SEQ project as described in ref. 2. Family M was targeted resequencing as described in ref. 3. Families J and T were targeted sequenced as described in ref. 4. Whole exome sequenced at the Genomics Platform at the Broad Institute of MIT and Harvard Center for Mendelian Genomics (Broad CMG, Cambridge, MA, USA) using a 38-Mb targeted Twist exome capture was carried out for families L and S (as trios), O (as quad) and P (as singleton). Family Q was whole genome sequencing at the Broad CMG; PCR-free preparation of sample DNA (350 ng input at >2 ng/uL) was accomplished using Illumina HiSeq X Ten v2 chemistry. Families H and U were whole exome sequenced using an Agilent SureSelect Human All Exon 50 Mb Kit (Santa Clara, CA, USA) as described in ref. 5. Family N was whole exome sequenced using an Agilent SureSelect XT (V6 Panel, Santa Clara, CA, USA). Family G was whole exome sequenced by GeneDx (Gaithersberg, MD, USA).

Segregation analysis

When DNA from family members was available, segregations were carried out by standard PCR amplification and Sanger sequencing, using the following primers:

	SRPK3 variant(s)	*TTN* variant(s)
Fam D	Fwd 5'- TGTGGCCCTCAAAGTGGT<br>Rev 5'- CTCGCCTGTTCCTCCAGAT	Fwd 5'- CTCTCTCAAGCACACCCACC<br>Rev 5'- ACTGCTTCATCCAGTGCCAA
Fam E	Fwd 5'- CAGGAGAAGGGTACACTGAAGG<br>Rev 5'- AATCAACGCTAGCTTGTTACTGC	Fwd 5'- CAGCAACAATTTTCCCAGAGTAA<br>Rev 5'- TATCTCTGACACTGGCATCTAGC
Fam M	Fwd 5'- GATGGTCAATGGGGCCGAGGT<br>Rev 5'- TGGAAGAAAGGCCGCCAACC<br>Fwd 5'- CACATCCTCTTCAGGCTGTCAC<br>Rev 5'- GTGCACTGCAGACTGGGGTAG	Fwd 5'- AGTGACAATCTGCCATTCATCTTC<br>Rev 5'- AGTACAGAAACGTGAAACCAGCAG
Fam O	na<br>na	Fwd 5'- GTAACCCTACACCCACCTCC<br>Rev 5'- AGGACAGCAGCTTCTCTCAG
Fam Q	Fwd 5'- CAAGCACTTCACGGAAGACA<br>Rev 5'- ACCCAGTGTTCCCTCTGTTG	Fwd 5'- AAATGGTCAGAGTGTGCTCG<br>Rev 5'- AACATCCCATACTGTGGTGG
Fam R	Fwd 5'- CTCCTGTCGCCTAGCAGTAAGT<br>Rev 5'- CTGAGGCTGATCCCTTCCTAGT	Fwd 5'- AACTTCTGTTATTGCCAAAGCTG<br>Rev 5'- GTTCTCTTGGTGGCATCTCTCT
Fam S	Fwd 5'- ACATCAAGCCCGAGAACATC<br>Rev 5'- AGCATCCTTTCACCAAGAGC	Fwd 5'- AAGGTAAATTTGCTTGGGTCA<br>Rev 5'- TGAGAGCAGAAAACAGATACGG
Fam V	Fwd 5'- CTTCAGGGCAGGGTGGAACCATCT<br>Rev 5'- TTTACTGGGAAGGATGCAAGCCCATTGTGC	Fwd 5'- CTGCCACCATCATGTTCAGG<br>Rev 5'- CTTGGAAAATCGATCAGCTCCA<br>Fwd 5'- GATTTCTTGGTGGGGATGGG<br>Rev 5'- TTAGCATTCCCAAAGCGGTC
Fam Y	Fwd 6'- TGGTAAGTTGGGTGGCAAGTGGTG<br>Rev 5'- CAGGACCAGTGCCCACCCAGCCGACGGT	Fwd 5'- CCATCGTGTTTGGGCTTAGG<br>Rev 5'- TTCCACAGCCACCACTAAGT
Fam Z	Fwd 5'- AAAGGCTGCAAGACATCTGCT<br>Rev 5'- GATCTTGATCTTATCTGCATTTTGG	Fwd 5'- TCATCTAAGGTGATTTGGCAGTATT<br>Rev 5'- CTACCTCAGGAAGCATTAGCAC

X-inactivation assay

DNA from obligate female carriers extracted from peripheral blood was subjected to an initial PCR to confirm that the patient was heterozygous for the CAG repeat in exon 1 of the androgen receptor gene using primers 5’- TCCAGAATCTGTTCCAGAGCGT (forward) and 5’- (FAM) GGCTGTGAAGGTTGCTGTTCCTCAT (reverse). Female DNA was then spiked with male DNA (2:1 ratio) to act as control for restriction digest. One half of the spiked DNA was digested overnight at 37°C with the methylation sensitive enzymes HhaI and HpaII. The digested spiked DNA and the

undigested spiked DNA were then used as templates in the androgen receptor (fluorescent) PCR. A PCR product should only be seen for an inactive (methylated) X chromosome, which cannot be digested by the enzymes, HhaI and HpaII, and can therefore act as a template for the PCR. The presence of the male allele in the digested sample suggests that digestion has been compromised. Following capillary electrophoresis, Genemarker (http://www.softgenetics.com/GeneMarker.html) was used to compare the peak areas of the PCR product of the digested and undigested spiked DNA and to give a percentage of X-inactivation for the patient.

Statistical analysis of variant frequencies

We observed 6 truncating *SRPK3* coding variants amongst a control population of 67,961 male exomes from gnomAD v2.1.1 (https://gnomad.broadinstitute.org/). Based on the "Allele Number" (total number of called high quality genotypes) at positions across *SRPK3* provided by gnomAD v2.1.1 - which should have a maximum possible value of 183,535 (corresponding to 57,787 females and 67,961 hemizygous males) - we estimated that, across *SRPK3*, we have on average a 87.5% probability of actually observing an allele in an individual at any given coding position, once one allows for the removal (quality filtering) of some allele calls (e.g. due to low sequencing coverage). If we assume that the true probability of a deleterious variant occurring in a male individual is π, then this implies that probability of a deleterious variant actually being observed in the individual is ~0.875π, assuming that there is a negligible chance of more than one deleterious *SRPK3* variant occurring in an individual.

The number of observed *SRPK3* coding variants amongst the control population of 67,961 male exomes is thus distributed binomially with "success" parameter $p=0.875\pi$. Using the conservative approach for constructing confidence intervals proposed by Agresti and Coull (1998), we add two successes and two failures to the data in comparison to what is used for the usual Wald confidence

interval. This results in an estimate of E(p)=8/67965 with

variance((E(p))=(8/67965)*(67957/67965)/67965, giving a resulting estimate of

E(π)=(8/67965)/0.875=0.000135, and

Variance(E(π))=[(8/67965)*(67957/67965)/67965]/{0.875^2}=2.262E-09

implying a 95% confidence interval for π of [4.131E-5, 0.000228].

In our patient population, we initially observed 5 deleterious *SRPK3* coding variants amongst 1170 males. Assuming that all variants present were identified, a similar calculation to that above suggests that the expected value of π in the male UK patient population is 7/1174 = 0.00596 with variance (7/1174)*(1167/1174)/1174 = 5.049E-06. This implies a 95% confidence interval for π in the male UK patient population of [0.00156, 0.0104]. This interval lies well above the confidence interval seen in controls, suggesting that the frequency of variants in cases is significantly higher than in controls. Moreover, if one assumes a similar (87.5%) probability of actually observing an allele at any given coding position as seen in gnomAD v2.1.1, then the expected value of π in the male UK patient population becomes (7/1174)/0.875 = 0.00681 with variance [(7/1174)*(1167/1174)/1174]/{0.875^2} = 6.594E-06. This implies a 95% confidence interval for π in the male UK patient population of [0.00178, 0.0118], which again lies well above the confidence interval seen in controls.

More straightforwardly, one can test for a difference in the binomial probabilities of a deleterious variant both existing and being observed in the patient population compared to the control population, without making any specific assumption about the observation rate. (If we assume that both populations have the *same* observation rate, then this effectively tests for a difference in frequency of deleterious variants between the patient population and the control population). Following the proposal of Agresti and Coull (1996) to add two successes and two failures to the data, we estimate E(p) as 7/1174 = 0.00596 in the patient population and as 8/67965 = 0.000118 in

the control population, generating a two-sided p-value of p=2.00E-41 or a one-sided p value of p=1.00E-41 (as calculated from https://www.socscistatistics.com/tests/ztest/default2.aspx).

None of the 6 male exomes from gnomAD harbouring truncating *SRPK3* coding variants also carried a *TTN* truncating variant. If we assume that any *TTN* variants present would be observed, this suggests that the probability of deleterious variants occurring at both *SRPK3* and *TTN* in a control male individual π_2 can be estimated as

$E(\pi_2)=(2/67965)/0.875=3.363E\text{-}05$, and

$\text{Variance}(E(\pi_2))=[(2/67965)*(67963/67965)/67965]/\{0.875^2\}=5.645E^{-10}$

implying a 95% confidence interval for π_2 of [0, 8.024E-05].

More conservatively, allowing for the fact that *TTN* variants may also be missed, we could assume the same rate of observation of alleles (87.5%) across *TTN* as *SRPK3*. In that case, the probability of deleterious variants occurring at both *SRPK3* and *TTN* in a control male individual π_2 can be estimated as

$E(\pi_2)=(2/67965)/(0.875\hat{}2)=3.844E^{-05}$, and

$\text{Variance}(E(\pi_2))=[(2/67965)*(67963/67965)/67965]/\{0.875^4\}=7.386E^{-10}$

implying a 95% confidence interval for π_2 of [0, 9.170E^{-05}].

Thus, this indicates that our observation of seeing 31 males, ascertained on the basis of presenting with a skeletal muscle myopathy, all having deleterious variants at both *SRPK3* and *TTN* can be considered highly unusual.

Statistical modelling of familial segregation

To quantify the degree to which our own observations supported digenic inheritance of causal variants at *SRPK3* and *TTN*, we adapted an approach proposed by Thompson et al. (2003) that was originally designed for the evaluation of causality of sequence variants for family data in the context of a single gene.

Thompson et al. (2003) proposed using the following likelihood ratio (or Bayes factor):

$$B = \frac{L(\boldsymbol{V}|\boldsymbol{P}, V_p, C = 1)}{L(\boldsymbol{V}|\boldsymbol{P}, V_p, C = 0)}$$

where ***P*** is the vector of disease phenotypes within the family, ***V*** is the vector of variant genotypes, V_p is the variant genotype of the proband, *C*=1 denotes the event that the variant is disease-causing and *C*=0 denotes the event that the variant is neutral. Thompson et al. (2003) noted that the likelihoods in the numerator and denominator can be computed using a standard two-allele model with a hypothetical susceptibility allele *A* (corresponding to all deleterious alleles) and a normal allele *a* (corresponding to all neutral alleles). They proposed modelling the hypothesis *C*=1 by treating the measured variant as a genetic marker allele that is both in complete linkage (recombination fraction θ=0) and complete linkage disequilibrium (linkage disequilibrium parameter *D*=1) with the hypothetical susceptibility allele *A*. They proposed modelling *C*=0 by assuming that the variant occurs and segregates independently of the disease, equivalent to the same model but with θ=0.5 and *D*=0. This leads to the formulation:

$$B = \frac{L(\boldsymbol{P}, \boldsymbol{V}|\theta{=}0, D = 1)}{L(\boldsymbol{P}, \boldsymbol{V}|\theta{=}0.5, D = 0)} \times \frac{L(\boldsymbol{P}, V_p|\theta{=}0.5, D = 0)}{L(\boldsymbol{P}, V_p|\theta{=}0, D = 1)}$$

where the first ratio (on the left) is similar to the antilogarithm of the LOD score for linkage between the variant and disease but with additional information arising from the fact that the variant is assumed to be in phase with the susceptibility allele, and the second ratio is a correction for the fact that the proband is known to carry the variant. Both of these likelihood ratios can be calculated using standard software for family-based linkage analysis, such as the LINKAGE package (Lathrop et al. 1984), provided the software is capable of modelling linkage disequilibrium.

The LINKAGE package has previously been extended to perform two-locus linkage analysis, testing for segregation at two unlinked loci (Lathrop and Ott 1990), but, to our knowledge, the resulting software implementation, TMLINK, has not been made widely available, and, moreover, its primary focus (testing the null hypothesis of no linkage at either of two loci) does not precisely match the hypotheses in which we are most interested. Another freely available two-locus linkage analysis package, GENEHUNTER-TWOLOCUS (Strauch et al. 2000) has a similar limitation and, furthermore, is not well suited for incorporating some of

the features of our own study. Specifically, it is not designed to incorporate X linked loci, linkage disequilibrium or varying liability classes (whereby different individuals can be assigned different genotype-specific penetrances). In addition, by using the Lander-Green algorithm (Lander and Green 1987; Kruglyak et al. 1995) rather than the Elston-Stewart algorithm (Elston and Stewart 1971), it is ill-suited for analysis of the largest of our families, as the calculation time increases exponentially with the number of individuals in a pedigree (and thus becomes computationally prohibitive).

Instead, we chose to use the (single-locus) MLINK program from the original LINKAGE package to perform the calculations, taking advantage of its ability to model varying liability classes in order to allow us to evaluate the evidence for causality at *SRPK3* when you do or do not require deleterious variants to also be present at *TTN*, and vice versa. This formulation in terms of liability classes also allowed us to model some of the additional complicating features seen in our data set including possible X-inactivation (XI) in females heterozygous for variants at *SRPK3*, and the existence of one family that appeared to require two separate *TTN* variants (in addition to a *SRPK3* variant) for an individual to exhibit the disease. Rather than using the product of two likelihood ratios (with the second providing a correction for the fact that the proband is known to carry the variant), as was done by Thompson et al (2003), we instead chose to use only the first likelihood ratio on the left:

$$\frac{L(\boldsymbol{P},\boldsymbol{V}|\theta{=}0, D = 1)}{L(\boldsymbol{P},\boldsymbol{V}|\theta{=}0.5, D = 0)}$$

Our justification for not correcting for the fact that the proband carries the variant is because we wish to *specifically use* this information to provide additional strength for the evidence of causality. (In the context of sequencing studies, individual patients carrying a rare mutation are generally considered to add to the weight of evidence for its pathogenicity, over and above the evidence provided by the segregation of the mutation with disease phenotype in a pedigree). For comparison, we also present results based on the product of the two likelihood ratios (i.e. incorporating the correction for the fact that the proband is known to carry the variant).

For each gene, we defined up to 8 separate analysis models that corresponded to the combination of an assumed penetrance model and a full or reduced data set (specifically, with or without the inclusion of

family 19 (Fam V, in the main text), which was the family that appeared to require two separate *TTN* variants - in addition to a *SRPK3* variant - for an individual to exhibit the disease). Models 1, 2 and 3 were each divided into two sub-models, (a) and (b), that made different assumptions about the modelled penetrances for the *SRPK3* genotype for individuals that were not genotyped at *TTN* (whose *TTN* genotype-specific liability class for *SRPK3* was therefore undefined), and, similarly, about the modelled penetrances for the *TTN* genotype for individuals that were not genotyped at *SRPK3* (whose *SRPK3* genotype-specific liability class for *TTN* was therefore undefined). Each analysis model (and sub-model) was applied either assuming complete LD between the underlying susceptibility allele *A* and the relevant measured *SRPK3* or *TTN* allele, or assuming no LD between the underlying susceptibility allele *A* and the relevant measured *SRPK3* or *TTN* allele.

In sub-model (a) we assumed relatively high penetrances, reflecting the fact that variants at the gene defining the liability class were known to be segregating in the pedigree. For example, we assumed a *TTN* penetrance of 0.5 for a male carrying the *TTN* variant, reflecting the assumption that *SRPK3* variants are known to be segregating in the pedigree and thus, at least for simple pedigrees with at least two founders, one might expect a probability of around 0.5 that any particular chosen founder carries the *SRPK3* variant, allowing the effect of any *TTN* variant possessed to be visible. In sub-model (b) we assumed relatively low penetrances, reflecting the fact that variants at the gene defining the liability class are rare in the population. For example, we assumed a *TTN* penetrance of 0.01 for a male carrying the *TTN* variant, reflecting the assumption that *SRPK3* variants are rare in the population and so, in the absence of a *SRPK3* variant, possession of a *TTN* variant is unlikely to have any observable effect. We considered these two sub-models to represent the extremes of the possible unknown penetrance values at the test gene for individuals that were not genotyped at the liability class defining gene. In the end (see later) the results obtained were found to be relatively insensitive to the choice of sub-model (a) or (b).

The liability class definitions, assumed penetrances and data sets analysed for *SRPK3* and *TTN* for the different analysis models are listed in Additional Tables 1 and 2. When analysing *SRPK3*, possible XI in females heterozygous for variants at *SRPK3* was modelled by assigning females with a *TTN* variant and

measured random XI very low penetrance (as the non-mutant *SRPK3* allele would compensate for the effect of the mutant *SRPK3* allele), while females not genotyped at *TTN*, or with a *TTN* variant and skewed or non-informative XI, were assigned half the *TTN* penetrance of equivalent (not genotyped/genotyped) males. When analysing *TTN*, possible XI in females heterozygous for variants at *SRPK3* was modelled by assuming *TTN* variants would have very low penetrance in females with measured random XI (as the non-mutant *SRPK3* allele would compensate for the effect of the mutant *SRPK3* allele), while females not genotyped at *SRPK3*, or with an *SRPK3* variant and skewed or non-informative XI, were assigned half the *TTN* penetrance of equivalent (not genotyped/genotyped) males.

Results in terms of the LOD scores obtained when analysing *SRPK3* and *TTN* under the different analysis models are shown in Additional Tables 3 and 4, respectively. In both cases, the strongest evidence of linkage is obtained assuming complete LD and under analysis models 3a and 3b (which require both *SRPK3* and *TTN* variants to be present, and which include family 19 under a complex model requiring genotyped members of family 19 to have an additional *TTN* present to exhibit the disease). Highly significant maximum LOD scores of 10.47 (for *SRPK3*) or 11.86 (for *TTN*) are obtained at a recombination fraction θ=0 between the hypothetical susceptibility locus and the genotyped marker. In both cases, models 3a and 3b give slightly higher evidence of linkage than models 1a and 1b (which use the same underlying penetrance model but remove family 19 from the calculation) or models 2a and 2b (which use the same underlying penetrance model and include family 19, but without requiring genotyped members of family 19 to have an additional *TTN* present to exhibit the disease). Models 3a and 3b also give stronger evidence of linkage than any of models 4-8 (which assume that only the test locus - *SRPK3* or *TTN* - is important), although note that models 7 and 8, which allow for reduced penetrance at the test locus (without specifically modelling the effect of the other locus), still give what would be conventionally considered ``significant'' evidence of linkage (LOD>3, with a maximum likelihood estimate of the recombination fraction θ=0), provided complete LD is assumed between the hypothetical susceptibility locus and the genotyped marker.

In general, the level of evidence for linkage obtained is considerably stronger when assuming complete LD than when assuming no LD between the susceptibility locus and the genotyped marker, indicating the better

fit of (and greater power for testing linkage under) a model that treats the variant as a genetic marker allele that is in complete LD with the susceptibility allele A. Under complete LD, models 4, 5 and 6, which do not allow for reduced penetrance at the test locus, achieve their maximum LOD scores at θ>0, with lower evidence (and in some cases negative LOD scores) obtained at θ=0. These results are consistent with the observation that a fully penetrant model operating at the test locus alone is not a good fit for the data; in order to accommodate this assumed model, the possibility of recombination between alleles at the hypothetical susceptibility locus and the genotyped marker has to be invoked.

For models 1-3, at any given value of the recombination fraction, the differences in LOD score between the two sub-models, (a) and (b), that make different assumptions about the modelled penetrances for the *SRPK3* genotype for individuals that were not genotyped at *TTN*, and, similarly, about the modelled penetrances for the *TTN* genotype for individuals that were not genotyped at *SRPK3*, were seen to be negligible, indicating that our results are relatively insensitive to the choice of these assumptions.

The results shown in Additional Tables 3 and 4 illustrate that the greatest evidence for linkage is obtained when using the full data set (including family 19) and when invoking a complex model that involves both *SRPK3* and *TTN* variants. The primary purpose of LINKAGE package is to test for linkage (i.e. evidence in favour of θ<0 compared to θ=0.5), via the calculation of LOD scores (i.e. log base 10 of likelihood ratios). However, these LOD scores are not ideally suited for comparing *across* different analysis models, as the denominators of the relevant likelihood ratios (corresponding to the likelihoods of the data at θ=0.5) are not the same across the different models, on account of the different assumptions made and the different data (with/without family 19) being used in the likelihood.

To allow direct comparison across analysis models, Additional Tables 5 and 6 show the results in terms of the log base 10 likelihoods (with/without inclusion of family 19) when testing *SRPK3*, while Additional Tables 7 and 8 show the equivalent results when testing *TTN*. The higher the $\log_{10}$ likelihood, the better supported the model. For *SRPK3*, the best supported model is 3a (when including family 19, Additional Table 5) or 1a (when not including family 19, Additional Table 6) at θ=0. For *TTN*, the best supported model is 3b (when including family 19, Additional Table 7) or 1b (when not including family 19, Additional Table 8) at θ=0.

Within each table, to compare different models, the relative support provided by the data can be obtained simply by subtracting the relevant entries from one another and calculating 10 to the power of this quantity. For example, in Additional Table 5, focussing on the results obtained under complete LD at the maximum likelihood estimate of the recombination fraction (θ=0), we see that the data is $10^{(-99.899-(-100.898))}$ ≈10 times more likely under model 3a than under model 3b, and $10^{(-99.899-(-124.968))}$ ≈10^{25} times more likely under model 3a than under model 5. We also see that, at θ=0, the data is $10^{(-99.899-(-178.184))}$ ≈10^{78} times more likely under model 3a assuming complete LD than it is under model 3a assuming no LD.

In general, across Additional Tables 5-8, for any analysis model and any value of θ, the data is considerably more likely under (and thus more supportive of) a scenario of complete LD than a scenario of no LD. Assuming complete LD, the best supported models (3a and 1a for *SRPK3*, and 3b and 1b for *TTN*), which assume that variants at both *SRPK3* and *TTN* are important, are considerably better supported by the data (which is anywhere between approximately 10^{10} and 10^{80} times more likely) than models 4-8, which assume that only the test locus - *SRPK3* or *TTN* - is important.

As shown in Additional Tables 5 and 7, the best supported models (3a or 3b) when family 19 is included are those that model the effect of an alternative *TTN* variant in family 19, generating likelihoods that are typically 1000 times greater than the equivalent model (2a or 2b) that does not model the effect of an alternative *TTN* variant. The likelihoods for the best supported models are also seen to be approximately 10 times greater than those of their alternative sub-model (3b or 3a, respectively). Similarly (see Additional Tables 6 and 8), the best supported models (1a or 1b) when family 19 is not included also generate likelihoods that are typically 10 times greater than their alternative sub-model (1b or 1a, respectively). Overall, the best performing models are seen to be those that model the various complicating features seen in our data set, including possible XI in females heterozygous for variants at *SRPK3*, and the existence of one family (family 19 or V) that requires two separate *TTN* variants (in addition to a *SRPK3* variant) for an individual to exhibit the disease. To assess the importance of modelling XI, we constructed alternative versions of models 1a, 1b, 3a and 3b, for use when testing *TTN*, that took into account the effect of variants at *SRPK3*, but not any possible XI in relation to these variants, in the *TTN* penetrances assumed. Specifically,

three liability classes were used. Genotyped individuals with no *SRPK3* variants, or genotyped members of family 19 without an additional *TTN* variant, were assigned *TTN* penetrances (0.0001, 0.0001, 0.0001). Individuals possessing a *SRPK3* variant (and, if a member of family 19, an additional *TTN* variant) were assigned *TTN* penetrances (0.0001, 0.9999, 0.9999). Individuals who were not genotyped at *SRPK3* were assigned *TTN* penetrances (0.0001, 0.5, 0.5) (sub-model (a)) or (0.0001, 0.01, 0.01) (sub-model (b)). Results are shown in Additional Tables 9 and 10. We see that the data is considerably more supportive (around 10^{20} times more likely when assuming complete LD, or 10^{12} times more likely when assuming no LD) of the models that incorporate possible XI.

The reduced penetrance models that we considered (models 7 and 8) – which assumed that only the test locus (*SRPK3* or *TTN*) – was important, assumed 50% penetrance (or 25% penetrance for *SRPK3* for females with skewed or non-informative XI). To check that our results were not too sensitive to this assumed value, we repeated the analysis of model 8 (including family 19) using a grid of penetrance values varied from 0.1 to 0.9 (with the penetrance for *SRPK3* halved for females with skewed or non-informative XI). Results for these models (labelled 8a to 8i, respectively, with 8e corresponding to the previous model 8) are shown in Additional Tables 11 and 12. Although in some cases a slightly better fit to the data is achieved using a penetrance value different from 0.5 (e.g. a penetrance of 0.6 or 0.7 for *SRPK3*, or 0.4 for *TTN*), the main observation, namely that a better fit is achieved using model 3 – which requires variants at both *SRPK3* and *TTN* for disease development – remains true, with the likelihood of the data seen to be at least 10^{10} times higher under model 3 than under any of the versions of model 8.

The specific likelihood rati– proposed by Thompson et al. (2003) for assessing causality of a sequence variant is:

$$B = \frac{L(\boldsymbol{P}, \boldsymbol{V}|\theta{=}0, D = 1)}{L(\boldsymbol{P}, \boldsymbol{V}|\theta{=}0.5, D = 0)} \times \frac{L(\boldsymbol{P}, V_p|\theta{=}0.5, D = 0)}{L(\boldsymbol{P}, V_p|\theta{=}0, D = 1)}$$

or, equivalently:

$$\frac{L(\boldsymbol{P}, \boldsymbol{V}|\theta{=}0, D = 1)}{L(\boldsymbol{P}, \boldsymbol{V}|\theta{=}0.5, D = 0)} \Big/ \frac{L(\boldsymbol{P}, V_p|\theta{=}0, D = 1)}{L(\boldsymbol{P}, V_p|\theta{=}0.5, D = 0)}$$

while we propose instead using:

$$\frac{L(\boldsymbol{P},\boldsymbol{V}|\theta=0, D = 1)}{L(\boldsymbol{P},\boldsymbol{V}|\theta=0.5, D = 0)}$$

Both of these quantities can be calculated using entries that appear in Additional Tables 5-8. (Note that the likelihoods shown in the final column of Additional Tables 5-8, required to calculate the likelihood ratio of Thompson et al. (2003), are calculated using the best-performing analysis model from the previous columns, but with genotype data at the test locus for all individuals except the proband in each family set to be missing). The relevant entries for calculating our proposed likelihood ratio are marked with a bold (§) while for the likelihood ratio of Thompson et al. (2003) we additionally make use of the terms marked with a bold (*). Thus, the likelihood ratio in favour of causality for *SRPK3*, when data from family 19 is included (Additional Table 5), is seen to be $10^{(-99.899-(-181.745))} \approx 10^{81.846}$ using our approach or $[10^{(-99.899-(-181.745))}]/[10^{(-93.475-(-171.207))}] \approx 10^{4.114}$ using the approach of Thompson et al. (2003). On the log to base 10 scale, this corresponds to $\log_{10}$ likelihoods in favour of causality of 81.846 using our approach or 4.117 using the approach of Thompson et al. (2003). Both of these clearly represent compelling evidence for causality, with the stronger evidence provided by our approach attributable to the fact that we choose not to correct for the fact that the proband is known to carry the variant, but rather consider this as additional evidence that should be included in the calculation.

Similar calculations applied to the relevant entries of Additional Tables 6-8 indicate that the $\log_{10}$ likelihoods in favour of causality for *SRPK3* when data from family 19 is not included (Additional Table 6) are 78.089 using our approach or 3.989 using the approach of Thompson et al. (2003); the $\log_{10}$ likelihoods in favour of causality for *TTN* when data from family 19 is included (Additional Table 7) are 79.886 using our approach or 5.774 using the approach of Thompson et al. (2003); and the $\log_{10}$ likelihoods in favour of causality for *TTN* when data from family 19 is not included (Additional Table 8) are 76.131 using our approach or 5.350 using the approach of Thompson et al. (2003). As expected, slightly greater evidence of causality is obtained when family 19 is included (and modelled appropriately) and considerably greater evidence of causality is obtained when evidence from the probands is included in the calculation.

We focussed in Additional Tables 5-12 on calculating likelihoods that either assumed complete LD, or else no LD, between the susceptibility locus and the genotyped marker, as these assumptions allowed us to calculate the specific likelihood ratio in favour of causality proposed by Thompson et al. (2003) (as well as our own modified version). To explore whether intermediate levels of LD might provide a better fit to the data, we used the PSEUDOMARKER program (Hiekkalinna et al. 2011; Gertz et al. 2014), which implements the likelihood-based approach proposed by Göring and Terwilliger (2000) that maximises over both the recombination fraction θ and the level of linkage disequilibrium (parameterised using a parameter denoted as δ). PSEUDOMARKER was applied assuming the best performing models (1a and 3a for *SRPK3*, 1b and 3b for *TTN*) from Additional Tables 5-8.

The results from the PSEUDOMARKER analysis are shown in Additional Table 13. At both *SRPK3* and *TTN* we find highly significant evidence of both linkage and LD between the susceptibility locus and the genotyped marker, with the strongest evidence being obtained when the other phenomenon is allowed for (i.e. when testing linkage given LD or LD given linkage). Under PSEUDOMARKER hypothesis H3, which corresponds to the existence of both linkage and LD, the maximum likelihood estimates of the linkage disequilibrium parameters do indeed correspond to a situation of complete LD ($D'=r^2=1$), supporting the notion that the variant allele at the genetic marker is in fact the hypothetical susceptibility allele *A*, i.e. the variant allele at the genetic marker is indeed the causal allele.

Model	Description of model	Family 19 included?	Liability Class	Penetrances of *SRPK3* genotypes (*aa*, *aA*, *AA*) in females	Penetrances of *SRPK3* genotypes (*a*, *A*) in males	Description of individuals assigned to this liability class
1a	A *TTN* variant needs to be present for *SRPK3* to have non-negligible penetrance. Heterozygous females not genotyped at *TTN,* or with an *TTN* variant and skewed or non-informative XI, are assigned half the *SRPK3* penetrance of equivalent males.	No	1	(0.0001, 0.0001, 0.0001)	(0.0001, 0.0001)	No *TTN* variants present
			2	(0.0001, 0.5, 0.9999)	(0.0001, 0.9999)	Males with an *TTN* variant, or females with a *TTN* variant and skewed or non-informative XI
			3	(0.0001, 0.25, 0.5)	(0.0001, 0.5)	Not genotyped at *TTN*
			4	(0.0001, 0.0001, 0.9999)	NA, designate as (0.0001, 0.0001)	Females with a *TTN* variant and random XI
1b	A *TTN* variant needs to be present for *SRPK3* to have non-negligible penetrance. Heterozygous females not genotyped at *TTN,* or with an *TTN* variant and skewed or non-informative XI, are assigned half the *SRPK3* penetrance of equivalent males.	No	1	(0.0001, 0.0001, 0.0001)	(0.0001, 0.0001)	No *TTN* variants present
			2	(0.0001, 0.5, 0.9999)	(0.0001, 0.9999)	Males with an *TTN* variant, or females with a *TTN* variant and skewed or non-informative XI
			3	(0.0001, 0.005, 0.01)	(0.0001, 0.01)	Not genotyped at *TTN*
			4	(0.0001, 0.0001, 0.9999)	NA, designate as (0.0001, 0.0001)	Females with a *TTN* variant and random XI
2a	A *TTN* variant needs to be present for *SRPK3* to have non-negligible penetrance. Heterozygous females not genotyped at *TTN,* or with an *TTN* variant and skewed or non-informative XI, are assigned half the *SRPK3* penetrance of equivalent males.	Yes	1	(0.0001, 0.0001, 0.0001)	(0.0001, 0.0001)	No *TTN* variants present
			2	(0.0001, 0.5, 0.9999)	(0.0001, 0.9999)	Males with an *TTN* variant, or females with a *TTN* variant and skewed or non-informative XI
			3	(0.0001, 0.25, 0.5)	(0.0001, 0.5)	Not genotyped at *TTN*
			4	(0.0001, 0.0001, 0.9999)	NA, designate as (0.0001, 0.0001)	Females with a *TTN* variant and random XI
2b	A *TTN* variant needs to be present for *SRPK3* to have non-negligible penetrance. Heterozygous females not genotyped at *TTN,* or with an *TTN* variant and skewed or non-informative XI, are assigned half the *SRPK3* penetrance of equivalent males.	Yes	1	(0.0001, 0.0001, 0.0001)	(0.0001, 0.0001)	No *TTN* variants present
			2	(0.0001, 0.5, 0.9999)	(0.0001, 0.9999)	Males with an *TTN* variant, or females with a *TTN* variant and skewed or non-informative XI
			3	(0.0001, 0.005, 0.01)	(0.0001, 0.01)	Not genotyped at *TTN*
			4	(0.0001, 0.0001, 0.9999)	NA, designate as (0.0001, 0.0001)	Females with a *TTN* variant and random XI
3a	A *TTN* variant needs to be present for *SRPK3* to have non-negligible penetrance.	Yes	1	(0.0001, 0.0001, 0.0001)	(0.0001, 0.0001)	No *TTN* variants present, or a member of family 19 with no additional *TTN* variant present.

	Heterozygous females not genotyped at *TTN,* or with an *TTN* variant and skewed or non-informative XI, are assigned half the *SRPK3* penetrance of equivalent males. Genotyped members of family 19 need an additional *TTN* variant to be present for *SRPK3* to have non-negligible penetrance.		2	(0.0001, 0.5, 0.9999)	(0.0001, 0.9999)	Males with an *TTN* variant, or females with a *TTN* variant and skewed or non-informative XI. (And, if a member of family 19, also with an additional *TTN* variant).
			3	(0.0001, 0.25, 0.5)	(0.0001, 0.5)	Not genotyped at *TTN*
			4	(0.0001, 0.0001, 0.9999)	NA, designate as (0.0001, 0.0001)	Females with a *TTN* variant and random XI. (And, if a member of family 19, also with an additional *TTN* variant).
3b	A *TTN* variant needs to be present for *SRPK3* to have non-negligible penetrance. Heterozygous females not genotyped at *TTN,* or with an *TTN* variant and skewed or non-informative XI, are assigned half the *SRPK3* penetrance of equivalent males. Genotyped members of family 19 need an additional *TTN* variant to be present for *SRPK3* to have non-negligible penetrance.	Yes	1	(0.0001, 0.0001, 0.0001)	(0.0001, 0.0001)	No *TTN* variants present, or a member of family 19 with no additional *TTN* variant present.
			2	(0.0001, 0.5, 0.9999)	(0.0001, 0.9999)	Males with an *TTN* variant, or females with a *TTN* variant and skewed or non-informative XI. (And, if a member of family 19, also with an additional *TTN* variant).
			3	(0.0001, 0.005, 0.01)	(0.0001, 0.01)	Not genotyped at *TTN*
			4	(0.0001, 0.0001, 0.9999)	NA, designate as (0.0001, 0.0001)	Females with a *TTN* variant and random XI. (And, if a member of family 19, also with an additional *TTN* variant).
4	Only *SRPK3* is important. A *TTN* variant is not required for *SRPK3* to have non-negligible penetrance.	No	1	(0.0001, 0.0001, 0.9999)	NA, designate as (0.0001, 0.0001)	Females with random XI.
			2	(0.0001, 0.5, 0.9999)	(0.0001, 0.9999)	Males, or females with skewed or non-informative XI.
5	Only *SRPK3* is important. A *TTN* variant is not required for *SRPK3* to have non-negligible penetrance.	Yes	1	(0.0001, 0.0001, 0.9999)	NA, designate as (0.0001, 0.0001)	Females with random XI.
			2	(0.0001, 0.5, 0.9999)	(0.0001, 0.9999)	Males, or females with skewed or non-informative XI.
7	Only *SRPK3* is important. A *TTN* variant is not required for *SRPK3* to have non-negligible penetrance.	No	1	(0.0001, 0.0001, 0.5)	NA, designate as (0.0001, 0.0001)	Females with random XI.
			2	(0.0001, 0.25, 0.5)	(0.0001, 0.5)	Males, or females with skewed or non-informative XI.
8	Only *SRPK3* is important. A *TTN* variant is not required for *SRPK3* to have non-negligible penetrance.	Yes	1	(0.0001, 0.0001, 0.5)	NA, designate as (0.0001, 0.0001)	Females with random XI.
			2	(0.0001, 0.25, 0.5)	(0.0001, 0.5)	Males, or females with skewed or non-informative XI.

Additional Table 1: *SRPK3* penetrance models

Model	Description of model	Family 19 included?	Liability Class	Penetrances of *TTN* genotypes (***aa***, ***aA***, ***AA***)	Description of individuals assigned to this liability class
1a	A *SRPK3* variant needs to be present for *TTN* to have non-negligible penetrance. Females not genotyped at *SRPK3,* or with an *SRPK3* variant and skewed or non-informative XI, are assigned half the *TTN* penetrance of equivalent males.	No	1	(0.0001, 0.0001, 0.0001)	No *SRPK3* variants present, or females with an *SRPK3* variant and random XI
			2	(0.0001, 0.9999, 0.9999)	Males with an *SRPK3* variant
			3	(0.0001, 0.5, 0.5)	Males not genotyped at *SRPK3,* or females with an *SRPK3* variant and skewed or non-informative XI
			4	(0.0001, 0.25, 0.25)	Females not genotyped at *SRPK3*
1b	A *SRPK3* variant needs to be present for *TTN* to have non-negligible penetrance. Females not genotyped at *SRPK3,* or with an *SRPK3* variant and skewed or non-informative XI, are assigned half the *TTN* penetrance of equivalent males.	No	1	(0.0001, 0.0001, 0.0001)	No *SRPK3* variants present, or females with an *SRPK3* variant and random XI
			2	(0.0001, 0.9999, 0.9999)	Males with an *SRPK3* variant
			3	(0.0001, 0.01, 0.01)	Males not genotyped at *SRPK3*
			4	(0.0001, 0.005, 0.005)	Females not genotyped at *SRPK3*
			5	(0.0001, 0.5, 0.5)	Females with an *SRPK3* variant and skewed or non-informative XI
2a	A *SRPK3* variant needs to be present for *TTN* to have non-negligible penetrance. Females not genotyped at *SRPK3,* or with an *SRPK3* variant and skewed or non-informative XI, are assigned half the *TTN* penetrance of equivalent males.	Yes	1	(0.0001, 0.0001, 0.0001)	No *SRPK3* variants present, or females with an *SRPK3* variant and random XI
			2	(0.0001, 0.9999, 0.9999)	Males with an *SRPK3* variant
			3	(0.0001, 0.5, 0.5)	Males not genotyped at *SRPK3*, or females with an *SRPK3* variant and skewed or non-informative XI
			4	(0.0001, 0.25, 0.25)	Females not genotyped at *SRPK3*
2b	A *SRPK3* variant needs to be present for *TTN* to have non-negligible penetrance. Females not genotyped at *SRPK3,* or with an *SRPK3* variant and skewed or non-informative XI, are assigned half the *TTN* penetrance of equivalent males.	Yes	1	(0.0001, 0.0001, 0.0001)	No *SRPK3* variants present, or females with an *SRPK3* variant and random XI
			2	(0.0001, 0.9999, 0.9999)	Males with an *SRPK3* variant
			3	(0.0001, 0.01, 0.01)	Males not genotyped at *SRPK3*
			4	(0.0001, 0.005, 0.005)	Females not genotyped at *SRPK3*
			5	(0.0001, 0.5, 0.5)	Females with an *SRPK3* variant and skewed or non-informative XI
3a	A *SRPK3* variant needs to be present for *TTN* to have non-negligible penetrance. Females not genotyped at *SRPK3,* or with an *SRPK3* variant and skewed or non-informative XI, are assigned half the *TTN* penetrance of males not genotyped at *SRPK3.* Genotyped members of family 19 need an additional *TTN* variant to be present for the primary tested *TTN* variant to have non-negligible penetrance.	Yes	1	(0.0001, 0.0001, 0.0001)	No *SRPK3* variants present, or females with an *SRPK3* variant and random XI, or a member of family 19 who does not possess an additional *TTN* variant.
			2	(0.0001, 0.9999, 0.9999)	Males with an *SRPK3* variant (and, if a member of family 19, also with an additional *TTN* variant).
			3	(0.0001, 0.5, 0.5)	Males not genotyped at *SRPK3* (or *TTN*), or females with an *SRPK3* variant and skewed or non-informative XI. (And, if a genotyped member of family 19, also with an additional *TTN* variant).
			4	(0.0001, 0.25, 0.25)	Females not genotyped at *SRPK3* (or *TTN*)
3b	A *SRPK3* variant needs to be present for *TTN* to have non-negligible penetrance. Females not genotyped at *SRPK3,* or with an *SRPK3*	Yes	1	(0.0001, 0.0001, 0.0001)	No *SRPK3* variants present, or females with an *SRPK3* variant and random XI, or a member of family 19 who does not possess an additional *TTN* variant.

	variant and skewed or non-informative XI, are assigned half the *TTN* penetrance of equivalent males. Genotyped members of family 19 need an additional *TTN* variant to be present for the primary tested *TTN* to have non-negligible penetrance.		2	(0.0001, 0.9999, 0.9999)	Males with an *SRPK3* variant (and, if a member of family 19, also with an additional *TTN* variant).
			3	(0.0001, 0.01, 0.01)	Males not genotyped at *SRPK3* (or *TTN*)
			4	(0.0001, 0.005, 0.005)	Females not genotyped at *SRPK3* (or *TTN*)
			5	(0.0001, 0.5, 0.5)	Females with an *SRPK3* variant and skewed or non-informative XI. (And, if a member of family 19, also with an additional *TTN* variant).
4	Only *TTN* is important. A *SRPK3* variant is not required for *TTN* to have non-negligible penetrance.	No	1	(0.0001, 0.9999, 0.9999)	All individuals are assigned to this liability class.
5	Only *TTN* is important. A *SRPK3* variant is not required for *TTN* to have non-negligible penetrance. Members of family 19 do not need an additional *TTN* variant to be present for the primary tested *TTN* to have non-negligible penetrance.	Yes	1	(0.0001, 0.9999, 0.9999)	All individuals are assigned to this liability class.
6	Only *TTN* is important. A *SRPK3* variant is not required for *TTN* to have non-negligible penetrance. Members of family 19 need an additional *TTN* variant to be present for the primary tested *TTN* to have non-negligible penetrance.	Yes	1	(0.0001, 0.0001, 0.0001)	Individuals in family 19 who do not possess an additional *TTN* variant.
			2	(0.0001, 0.9999, 0.9999)	Individuals in family 19 who do possess an additional *TTN* variant, and all individuals who are not in family 19.
7	Only *TTN* is important, but with incomplete penetrance. A *SRPK3* variant is not required for *TTN* to have non-negligible penetrance.	No	1	(0.0001, 0.5, 0.5)	All individuals are assigned to this liability class.
8	Only *TTN* is important, but with incomplete penetrance. A *SRPK3* variant is not required for *TTN* to have non-negligible penetrance. Members of family 19 do not need an additional *TTN* variant to be present for the primary tested *TTN* to have non-negligible penetrance.	Yes	1	(0.0001, 0.5, 0.5)	All individuals are assigned to this liability class.

Additional Table 2: *TTN* penetrance models

		Analysis model									
LD model	Theta	1a	1b	2a	2b	3a	3b	4	5	7	8
Complete LD	0.00	10.043	10.052	8.067	8.076	**10.469**	**10.477**	-3.263	-4.532	8.902	8.793
	0.05	9.300	9.308	7.845	7.853	9.692	9.700	5.919	4.674	8.282	8.203
	0.10	8.516	8.523	7.520	7.527	8.873	8.880	6.488	5.367	7.616	7.562
	0.15	7.686	7.692	6.981	6.988	8.007	8.013	6.392	5.472	6.899	6.867
	0.20	6.806	6.811	6.304	6.309	7.089	7.094	5.985	5.272	6.129	6.114
	0.25	5.869	5.874	5.516	5.521	6.112	6.116	5.372	4.843	5.300	5.299
	0.30	4.868	4.872	4.628	4.632	5.068	5.072	4.596	4.220	4.407	4.415
	0.35	3.793	3.796	3.641	3.644	3.948	3.951	3.672	3.423	3.442	3.455
	0.40	2.634	2.636	2.549	2.551	2.741	2.743	2.603	2.458	2.395	2.409
	0.45	1.376	1.377	1.340	1.341	1.431	1.432	1.384	1.321	1.253	1.262
	0.50	0.000	0.000	0.000	0.000	0.000	0.000	0.000	0.000	0.000	0.000
No LD	0.00	3.502	3.502	3.231	3.427	3.560	3.560	-5.889	-5.904	2.824	2.795
	0.05	3.107	3.107	2.798	3.044	3.155	3.155	1.053	1.041	2.534	2.510
	0.10	2.708	2.708	2.368	2.656	2.746	2.746	1.492	1.482	2.229	2.210
	0.15	2.308	2.308	1.942	2.267	2.338	2.338	1.543	1.535	1.913	1.898
	0.20	1.911	1.911	1.527	1.880	1.933	1.933	1.430	1.424	1.589	1.579
	0.25	1.521	1.521	1.130	1.499	1.536	1.536	1.227	1.223	1.266	1.258
	0.30	1.145	1.145	0.765	1.130	1.154	1.155	0.972	0.969	0.949	0.944
	0.35	0.792	0.792	0.448	0.784	0.797	0.797	0.693	0.691	0.650	0.648
	0.40	0.474	0.474	0.203	0.471	0.477	0.477	0.417	0.417	0.383	0.382
	0.45	0.206	0.206	0.050	0.205	0.207	0.207	0.177	0.177	0.162	0.162
	0.50	0.000	0.000	0.000	0.000	0.000	0.000	0.000	0.000	0.000	0.000

Additional Table 3: LOD score results from different *SRPK3* penetrance models. Entries showing the greatest evidence of linkage are marked in bold.

		Analysis model										
LD model	Theta	1a	1b	2a	2b	3a	3b	4	5	6	7	8
Complete LD	0.00	11.258	11.261	9.455	9.459	**11.859**	**11.862**	1.896	1.895	-0.163	9.329	9.351
	0.05	10.429	10.432	9.140	9.143	10.985	10.988	2.216	2.215	1.780	8.788	8.829
	0.10	9.553	9.556	8.710	8.713	10.062	10.065	2.338	2.337	2.152	8.152	8.207
	0.15	8.626	8.629	8.060	8.063	9.086	9.089	2.267	2.266	2.206	7.435	7.500
	0.20	7.642	7.644	7.264	7.267	8.049	8.052	2.095	2.095	2.106	6.642	6.712
	0.25	6.593	6.595	6.348	6.351	6.944	6.947	1.858	1.858	1.909	5.772	5.842
	0.30	5.471	5.473	5.323	5.325	5.763	5.765	1.572	1.572	1.642	4.819	4.885
	0.35	4.266	4.268	4.185	4.187	4.493	4.495	1.243	1.243	1.316	3.777	3.835
	0.40	2.964	2.965	2.929	2.930	3.122	3.123	0.873	0.873	0.934	2.637	2.681
	0.45	1.549	1.550	1.540	1.541	1.631	1.632	0.461	0.461	0.497	1.383	1.408
	0.50	0.000	0.000	0.000	0.000	0.000	0.000	0.000	0.000	0.000	0.000	0.000
No LD	0.00	3.279	3.278	3.231	3.230	3.388	3.387	1.093	1.093	0.996	2.087	2.076
	0.05	2.844	2.844	2.798	2.797	2.935	2.934	0.937	0.937	0.861	1.921	1.911
	0.10	2.409	2.409	2.368	2.367	2.482	2.482	0.790	0.790	0.730	1.696	1.689
	0.15	1.978	1.977	1.942	1.942	2.034	2.034	0.648	0.648	0.603	1.439	1.433
	0.20	1.555	1.555	1.527	1.527	1.598	1.597	0.514	0.514	0.481	1.165	1.160
	0.25	1.151	1.151	1.130	1.130	1.181	1.181	0.386	0.386	0.364	0.886	0.883
	0.30	0.778	0.778	0.765	0.764	0.798	0.798	0.269	0.269	0.254	0.616	0.614
	0.35	0.456	0.456	0.448	0.448	0.467	0.467	0.164	0.164	0.156	0.372	0.371
	0.40	0.206	0.206	0.203	0.203	0.211	0.211	0.078	0.078	0.075	0.174	0.173
	0.45	0.051	0.051	0.050	0.050	0.052	0.052	0.021	0.021	0.020	0.044	0.044
	0.50	0.000	0.000	0.000	0.000	0.000	0.000	0.000	0.000	0.000	0.000	0.000

Additional Table 4: LOD score results from different *TTN* penetrance models. Entries showing the greatest evidence of linkage are marked in bold.

		Likelihood $L(\boldsymbol{P},\boldsymbol{V}\|\theta,D)$						Likelihood $L(\boldsymbol{P},V_p\|\theta,D)$
		Analysis model						Analysis model
LD model	Theta	2a	2b	3a	3b	5	8	3a
Complete LD	0.00	-102.899	-103.898	**-99.899 ***	-100.898	-124.968	-111.611	**-93.475 §**
	0.05	-103.121	-104.121	-100.676	-101.675	-115.763	-112.201	-93.945
	0.10	-103.447	-104.447	-101.495	-102.495	-115.069	-112.842	-94.441
	0.15	-103.985	-104.986	-102.361	-103.362	-114.965	-113.537	-94.965
	0.20	-104.663	-105.664	-103.279	-104.281	-115.164	-114.290	-95.520
	0.25	-105.450	-106.453	-104.256	-105.259	-115.593	-115.105	-96.110
	0.30	-106.338	-107.342	-105.300	-106.303	-116.216	-115.989	-96.742
	0.35	-107.325	-108.330	-106.419	-107.424	-117.013	-116.949	-97.419
	0.40	-108.418	-109.423	-107.627	-108.632	-117.978	-117.995	-98.150
	0.45	-109.626	-110.632	-108.936	-109.943	-119.116	-119.141	-98.944
	0.50	-110.966	-111.974	-110.368	-111.375	-120.436	-120.404	-99.814
No LD	0.00	-178.486	-180.085	-178.184	-179.784	-193.537	-185.521	-171.207
	0.05	-178.869	-180.469	-178.590	-180.190	-186.592	-185.805	-171.207
	0.10	-179.257	-180.857	-178.998	-180.598	-186.151	-186.106	-171.207
	0.15	-179.646	-181.245	-179.407	-181.006	-186.097	-186.418	-171.207
	0.20	-180.033	-181.633	-179.812	-181.411	-186.208	-186.737	-171.207
	0.25	-180.414	-182.014	-180.208	-181.808	-186.410	-187.057	-171.207
	0.30	-180.783	-182.382	-180.590	-182.190	-186.664	-187.371	-171.207
	0.35	-181.129	-182.729	-180.947	-182.547	-186.942	-187.668	-171.207
	0.40	-181.442	-183.042	-181.268	-182.867	-187.216	-187.933	-171.207
	0.45	-181.707	-183.307	-181.538	-183.137	-187.456	-188.153	-171.207
	0.50	-181.913	-183.513	***-181.745 ****	-183.344	-187.633	-188.315	***-171.207* §**

Additional Table 5: $\log_{10}$ likelihoods from different *SRPK3* penetrance models when data from family 19 is included. The relevant entries for calculating our proposed likelihood ratio are marked with a bold (*), while for the likelihood ratio of Thompson et al. (2003) we additionally make use of the terms marked with a bold (§). Terms appearing in the denominator of the relevant likelihood ratios are additionally marked in italic.

		Likelihood $L(\boldsymbol{P},\boldsymbol{V}\|\theta,D)$				Likelihood $L(\boldsymbol{P},V_p\|\theta,D)$
		Analysis model				Analysis model
LD model	Theta	1a	1b	4	7	1a
Complete LD	0.00	**-94.393 ***	-95.392	-116.462	-104.785	**-89.350 §**
	0.05	-95.136	-96.136	-107.280	-105.404	-89.798
	0.10	-95.921	-96.921	-106.711	-106.071	-90.270
	0.15	-96.750	-97.751	-106.806	-106.788	-90.769
	0.20	-97.630	-98.632	-107.213	-107.558	-91.298
	0.25	-98.567	-99.570	-107.826	-108.386	-91.861
	0.30	-99.568	-100.572	-108.603	-109.279	-92.462
	0.35	-100.643	-101.647	-109.527	-110.244	-93.107
	0.40	-101.802	-102.807	-110.595	-111.291	-93.804
	0.45	-103.060	-104.067	-111.814	-112.433	-94.561
	0.50	-104.436	-105.444	-113.199	-113.687	-95.389
No LD	0.00	-168.980	-170.580	-184.031	-176.035	-163.450
	0.05	-169.375	-170.975	-177.088	-176.325	-163.450
	0.10	-169.774	-171.373	-176.650	-176.630	-163.450
	0.15	-170.174	-171.773	-176.599	-176.947	-163.450
	0.20	-170.571	-172.171	-176.712	-177.270	-163.450
	0.25	-170.961	-172.561	-176.915	-177.594	-163.450
	0.30	-171.337	-172.937	-177.170	-177.910	-163.450
	0.35	-171.690	-173.290	-177.449	-178.209	-163.450
	0.40	-172.008	-173.607	-177.724	-178.476	-163.450
	0.45	-172.276	-173.875	-177.965	-178.697	-163.450
	0.50	***-172.482 ****	-174.082	-178.142	-178.859	***-163.450 §***

Additional Table 6: $\log_{10}$ likelihoods from different *SRPK3* penetrance models when family 19 is excluded. The relevant entries for calculating our proposed likelihood ratio are marked with a bold (*), while for the likelihood ratio of Thompson et al. (2003) we additionally make use of the terms marked with a bold (§). Terms appearing in the denominator of the relevant likelihood ratios are additionally marked in italic.

		Likelihood $L(\boldsymbol{P},\boldsymbol{V}|\theta,D)$							Likelihood $L(\boldsymbol{P},V_p|\theta,D)$
		Analysis model							Analysis model
LD model	Theta	2a	2b	3a	3b	5	6	8	3b
Complete LD	0.00	-103.378	-103.106	-100.378	**-100.106 §**	-182.943	-182.902	-117.483	**-90.526 ***
	0.05	-103.694	-103.421	-101.252	-100.980	-182.623	-180.959	-118.006	-91.029
	0.10	-104.124	-103.851	-102.175	-101.902	-182.501	-180.587	-118.628	-91.559
	0.15	-104.773	-104.501	-103.151	-102.879	-182.572	-180.533	-119.335	-92.119
	0.20	-105.570	-105.298	-104.188	-103.916	-182.743	-180.633	-120.123	-92.712
	0.25	-106.485	-106.213	-105.293	-105.021	-182.980	-180.829	-120.993	-93.342
	0.30	-107.511	-107.240	-106.474	-106.203	-183.266	-181.097	-121.949	-94.016
	0.35	-108.648	-108.377	-107.744	-107.473	-183.595	-181.423	-123.000	-94.738
	0.40	-109.905	-109.634	-109.115	-108.845	-183.965	-181.804	-124.154	-95.518
	0.45	-111.293	-111.024	-110.606	-110.336	-184.378	-182.241	-125.426	-96.364
	0.50	-112.834	-112.564	-112.237	-111.968	-184.838	-182.739	-126.835	-97.289
No LD	0.00	-176.324	-176.931	-175.998	-176.605	-212.675	-212.675	-186.860	-164.639
	0.05	-176.757	-177.363	-176.451	-177.058	-212.830	-212.810	-187.025	-164.639
	0.10	-177.187	-177.793	-176.904	-177.510	-212.978	-212.941	-187.248	-164.639
	0.15	-177.612	-178.219	-177.352	-177.958	-213.120	-213.067	-187.503	-164.639
	0.20	-178.028	-178.634	-177.789	-178.395	-213.254	-213.190	-187.776	-164.639
	0.25	-178.424	-179.030	-178.205	-178.811	-213.381	-213.307	-188.054	-164.639
	0.30	-178.790	-179.396	-178.589	-179.195	-213.499	-213.416	-188.323	-164.639
	0.35	-179.107	-179.713	-178.919	-179.525	-213.604	-213.515	-188.566	-164.639
	0.40	-179.352	-179.958	-179.175	-179.781	-213.690	-213.596	-188.763	-164.639
	0.45	-179.505	-180.110	-179.334	-179.940	-213.747	-213.651	-188.892	-164.639
	0.50	-179.555	-180.161	-179.386	***-179.992 §***	-213.768	-213.671	-188.937	***-164.639 ****

Additional Table 7: log_{10} likelihoods from different *TTN* penetrance models when data from family 19 is included. The relevant entries for calculating our proposed likelihood ratio are marked with a bold (§) while for the likelihood ratio of Thompson et al. (2003) we additionally make use of the terms marked with a bold (*). Terms appearing in the denominator of the relevant likelihood ratios are additionally marked in italic.

		Likelihood $L(\boldsymbol{P},\boldsymbol{V}\|\theta,D)$				Likelihood $L(\boldsymbol{P},V_p\|\theta,D)$
		Analysis model				Analysis model
LD model	Theta	1a	1b	4	7	1b
Complete LD	0.00	-94.871	**-94.599 ***	-174.436	-110.485	**-86.401 §**
	0.05	-95.701	-95.428	-174.116	-111.026	-86.882
	0.10	-96.576	-96.304	-173.994	-111.662	-87.388
	0.15	-97.503	-97.231	-174.065	-112.379	-87.923
	0.20	-98.488	-98.216	-174.236	-113.172	-88.490
	0.25	-99.536	-99.265	-174.473	-114.043	-89.093
	0.30	-100.658	-100.387	-174.760	-114.995	-89.737
	0.35	-101.863	-101.592	-175.088	-116.037	-90.427
	0.40	-103.165	-102.895	-175.458	-117.177	-91.172
	0.45	-104.580	-104.310	-175.871	-118.431	-91.981
	0.50	-106.129	-105.860	-176.332	-119.814	-92.865
No LD	0.00	-166.845	-167.451	-203.169	-177.392	-157.182
	0.05	-167.279	-167.885	-203.325	-177.559	-157.182
	0.10	-167.714	-168.321	-203.473	-177.783	-157.182
	0.15	-168.146	-168.752	-203.614	-178.040	-157.182
	0.20	-168.569	-169.175	-203.748	-178.315	-157.182
	0.25	-168.973	-169.579	-203.876	-178.594	-157.182
	0.30	-169.345	-169.951	-203.993	-178.864	-157.182
	0.35	-169.668	-170.274	-204.098	-179.108	-157.182
	0.40	-169.917	-170.523	-204.184	-179.306	-157.182
	0.45	-170.073	-170.678	-204.241	-179.435	-157.182
	0.50	-170.124	***-170.729 ****	-204.262	-179.480	***-157.182 §***

Additional Table 8: $\log_{10}$ likelihoods from different *TTN* penetrance models when family 19 is excluded. The relevant entries for calculating our proposed likelihood ratio are marked with a bold (*), while for the likelihood ratio of Thompson et al. (2003) we additionally make use of the terms marked with a bold (§). Terms appearing in the denominator of the relevant likelihood ratios are additionally marked in italic.

		Analysis model, while incorporating modelling of XI		Analysis model without modelling XI	
LD model	Theta	3a	3b	3a	3b
Complete LD	0.00	-100.378	-100.106	-122.632	-120.575
	0.05	-101.252	-100.980	-123.456	-121.384
	0.10	-102.175	-101.902	-124.317	-122.228
	0.15	-103.151	-102.879	-125.219	-123.114
	0.20	-104.188	-103.916	-126.166	-124.046
	0.25	-105.293	-105.021	-127.166	-125.031
	0.30	-106.474	-106.203	-128.225	-126.077
	0.35	-107.744	-107.473	-129.353	-127.193
	0.40	-109.115	-108.845	-130.563	-128.394
	0.45	-110.606	-110.336	-131.871	-129.694
	0.50	-112.237	-111.968	-133.297	-131.115
No LD	0.00	-175.998	-176.605	-188.840	-187.963
	0.05	-176.451	-177.058	-189.169	-188.274
	0.10	-176.904	-177.510	-189.494	-188.582
	0.15	-177.352	-177.958	-189.815	-188.887
	0.20	-177.789	-178.395	-190.131	-189.189
	0.25	-178.205	-178.811	-190.437	-189.483
	0.30	-178.589	-179.195	-190.726	-189.762
	0.35	-178.919	-179.525	-190.982	-190.011
	0.40	-179.175	-179.781	-191.186	-190.208
	0.45	-179.334	-179.940	-191.314	-190.332
	0.50	-179.386	-179.992	-191.356	-190.373

Additional Table 9: $\log_{10}$ likelihoods from different *TTN* penetrance models, with and without modelling X-inactivation (XI), when family 19 is included.

		Analysis model, while incorporating modelling of XI		Analysis model without modelling XI	
LD model	Theta	1a	1b	1a	1b
Complete LD	0.00	-94.871	-94.599	-117.125	-115.068
	0.05	-95.701	-95.428	-117.905	-115.832
	0.10	-96.576	-96.304	-118.719	-116.630
	0.15	-97.503	-97.231	-119.571	-117.466
	0.20	-98.488	-98.216	-120.466	-118.345
	0.25	-99.536	-99.265	-121.409	-119.274
	0.30	-100.658	-100.387	-122.408	-120.260
	0.35	-101.863	-101.592	-123.473	-121.313
	0.40	-103.165	-102.895	-124.614	-122.444
	0.45	-104.580	-104.310	-125.846	-123.669
	0.50	-106.129	-105.860	-127.189	-125.007
No LD	0.00	-166.845	-167.451	-179.635	-178.758
	0.05	-167.279	-167.885	-179.943	-179.048
	0.10	-167.714	-168.321	-180.248	-179.336
	0.15	-168.146	-168.752	-180.551	-179.623
	0.20	-168.569	-169.175	-180.851	-179.909
	0.25	-168.973	-169.579	-181.142	-180.189
	0.30	-169.345	-169.951	-181.419	-180.455
	0.35	-169.668	-170.274	-181.666	-180.694
	0.40	-169.917	-170.523	-181.862	-180.884
	0.45	-170.073	-170.678	-181.986	-181.004
	0.50	-170.124	-170.729	-182.026	-181.044

Additional Table 10: $\log_{10}$ likelihoods from different *TTN* penetrance models, with and without modelling X-inactivation (XI), when family 19 is excluded.

		Analysis model (penetrance)								
LD model	Theta	8a (0.1)	8b (0.2)	8c (0.3)	8d (0.4)	8e (0.5)	8f (0.6)	8g (0.7)	8h (0.8)	8i (0.9)
Complete LD	0.00	-127.960	-119.804	-115.598	-113.094	-111.611	-110.885	-110.837	-111.540	-113.309
	0.05	-128.630	-120.472	-116.254	-113.726	-112.201	-111.402	-111.225	-111.685	-112.947
	0.10	-129.336	-121.177	-116.950	-114.402	-112.842	-111.985	-111.712	-112.004	-112.962
	0.15	-130.081	-121.923	-117.689	-115.125	-113.537	-112.635	-112.287	-112.458	-113.209
	0.20	-130.872	-122.715	-118.476	-115.899	-114.290	-113.351	-112.947	-113.028	-113.634
	0.25	-131.713	-123.559	-119.316	-116.731	-115.105	-114.139	-113.692	-113.707	-114.210
	0.30	-132.611	-124.461	-120.218	-117.627	-115.989	-115.003	-114.525	-114.492	-114.923
	0.35	-133.574	-125.431	-121.188	-118.594	-116.949	-115.950	-115.451	-115.387	-115.771
	0.40	-134.613	-126.478	-122.238	-119.644	-117.995	-116.990	-116.480	-116.397	-116.754
	0.45	-135.742	-127.615	-123.380	-120.788	-119.141	-118.136	-117.622	-117.533	-117.880
	0.50	-136.975	-128.860	-124.631	-122.046	-120.404	-119.403	-118.895	-118.811	-119.164
No LD	0.00	-195.779	-190.991	-188.340	-186.640	-185.521	-184.830	-184.514	-184.604	-185.338
	0.05	-196.088	-191.303	-188.649	-186.941	-185.805	-185.083	-184.707	-184.674	-185.087
	0.10	-196.402	-191.620	-188.967	-187.253	-186.106	-185.361	-184.945	-184.840	-185.105
	0.15	-196.718	-191.942	-189.289	-187.573	-186.418	-185.658	-185.216	-185.065	-185.245
	0.20	-197.033	-192.262	-189.612	-187.896	-186.737	-185.968	-185.509	-185.330	-185.458
	0.25	-197.341	-192.578	-189.932	-188.217	-187.057	-186.284	-185.816	-185.619	-185.717
	0.30	-197.636	-192.881	-190.240	-188.529	-187.371	-186.598	-186.126	-185.921	-186.002
	0.35	-197.909	-193.163	-190.527	-188.822	-187.668	-186.897	-186.426	-186.219	-186.294
	0.40	-198.152	-193.412	-190.783	-189.083	-187.933	-187.167	-186.700	-186.496	-186.571
	0.45	-198.353	-193.619	-190.994	-189.298	-188.153	-187.392	-186.929	-186.729	-186.808
	0.50	-198.505	-193.775	-191.152	-189.458	-188.315	-187.556	-187.096	-186.899	-186.982

Additional Table 11: $\log_{10}$ likelihoods from different *SRPK3* reduced penetrance models with family 19 included.

		Analysis model (penetrance)								
LD model	Theta	8a (0.1)	8b (0.2)	8c (0.3)	8d (0.4)	8e (0.5)	8f (0.6)	8g (0.7)	8h (0.8)	8i (0.9)
Complete LD	0.00	-127.591	-120.625	-117.806	-116.952	-117.483	-119.302	-122.623	-128.072	-137.401
	0.05	-128.336	-121.354	-118.500	-117.583	-118.006	-119.634	-122.617	-127.469	-135.746
	0.10	-129.122	-122.128	-119.246	-118.281	-118.628	-120.142	-122.961	-127.604	-135.688
	0.15	-129.955	-122.950	-120.046	-119.044	-119.335	-120.769	-123.475	-127.970	-135.920
	0.20	-130.838	-123.827	-120.905	-119.875	-120.123	-121.496	-124.118	-128.503	-136.351
	0.25	-131.779	-124.764	-121.829	-120.777	-120.993	-122.320	-124.878	-129.180	-136.950
	0.30	-132.785	-125.769	-122.825	-121.757	-121.949	-123.242	-125.754	-129.994	-137.704
	0.35	-133.866	-126.851	-123.902	-122.824	-123.000	-124.269	-126.748	-130.944	-138.611
	0.40	-135.032	-128.021	-125.071	-123.987	-124.154	-125.410	-127.868	-132.036	-139.674
	0.45	-136.299	-129.295	-126.347	-125.262	-125.426	-126.677	-129.126	-133.280	-140.902
	0.50	-137.684	-130.691	-127.748	-126.667	-126.835	-128.088	-130.539	-134.693	-142.309
No LD	0.00	-193.966	-189.598	-187.620	-186.824	-186.860	-187.637	-189.239	-192.001	-196.730
	0.05	-194.279	-189.901	-187.901	-187.065	-187.025	-187.664	-189.002	-191.252	-195.214
	0.10	-194.592	-190.208	-188.193	-187.329	-187.248	-187.824	-189.079	-191.228	-195.097
	0.15	-194.901	-190.514	-188.488	-187.609	-187.503	-188.048	-189.265	-191.373	-195.203
	0.20	-195.200	-190.812	-188.782	-187.893	-187.776	-188.307	-189.508	-191.600	-195.416
	0.25	-195.482	-191.096	-189.065	-188.174	-188.054	-188.582	-189.781	-191.872	-195.688
	0.30	-195.737	-191.356	-189.328	-188.440	-188.323	-188.855	-190.060	-192.161	-195.988
	0.35	-195.953	-191.579	-189.556	-188.675	-188.566	-189.108	-190.325	-192.441	-196.286
	0.40	-196.117	-191.750	-189.735	-188.863	-188.763	-189.317	-190.548	-192.681	-196.546
	0.45	-196.217	-191.857	-189.849	-188.983	-188.892	-189.456	-190.699	-192.845	-196.726
	0.50	-196.250	-191.892	-189.887	-189.024	-188.937	-189.504	-190.752	-192.903	-196.790

Additional Table 12: $\log_{10}$ likelihoods from different *TTN* reduced penetrance models with family 19 included.

		P values					Maximum likelihood estimates of LD measures between disease and marker loci							
							Hypothesis H2: Linkage=No, LD=YES				Hypothesis H3: Linkage=YES, LD=YES			
**Gene**	**Model**	Linkage	LD|Linkage	LD|NoLinkage	Linkage|LD	LD+Linkage	δ	D'	r	r^2	δ	D'	r	r^2
SRPK3	1a	0.000104	5.288e-11	0.000091	1.173e-10	2.429e-13	-0.000083	-1.000	0.022376	0.000501	-0.000100	-1.000	1.000	1.000
	3a	0.000089	1.493e-11	0.000059	4.330e-11	6.093e-14	-0.000084	-1.000	0.022696	0.000515	-0.000100	-1.000	1.000	1.000
TTN	1b	0.000534	9.472e-13	0.000603	1.627e-12	2.161e-14	-0.000091	-1.000	0.032256	0.001040	-0.000100	-1.000	1.000	1.000
	3b	0.000400	2.168e-13	0.000411	4.078e-13	3.868e-15	-0.000091	-1.000	0.032545	0.001059	-0.000100	-1.000	1.000	1.000

Additional Table 13: Results from PSEUDOMARKER analysis.

References

1. Böhm J, Schneider R, Malfatti E et al. (2017) Integrated analysis of the large-scale sequencing project “Myocapture” to identify novel genes for myopathies. Neuromuscular Disorders. 27(2):S195.
2. Töpf A, Johnson K, Bates A, et al. (2020) Sequential targeted exome sequencing of 1001 patients affected by unexplained limb-girdle weakness. Genetics in Medicine 22:1478–1488.
3. Evila A, Arumilli M, Udd B, Hackman P (2016) Targeted next-generation sequencing assay for detection of mutations in primary myopathies. Neuromuscul Disord 26:7–15
4. Beecroft SJ, Yau KS, Allcock RJ, el al. (2020) Targeted gene panel use in 2249 neuromuscular patients: the Australasian referral center experience. Ann Clin Transl Neurol. 7(3):353–362
5. Neveling K, Feenstra I, Gilissen C, et al. (2013) A post-hoc comparison of the utility of sanger sequencing and exome sequencing for the diagnosis of heterogeneous diseases. Hum Mutat. 34(12):1721-6.
6. Agresti and Coull (1996) Order-restricted tests for stratified comparisons of binomial proportions." Biometrics 52(3): 1103-1111.
7. Elston RC, Stewart J (1971) A general model for the genetic analysis of pedigree data. Hum Hered 21:523–542
8. Gertz EM, Hiekkalinna T, Digabel SL, Audet C, Terwilliger JD, Schäffer AA (2014) PSEUDOMARKER 2.0: efficient computation of likelihoods using NOMAD. BMC Bioinformatics 15:47.
9. Göring HH, Terwilliger JD (2000) Linkage analysis in the presence of errors IV: joint pseudomarker analysis of linkage and/or linkage disequilibrium on a mixture of pedigrees and singletons when the mode of inheritance cannot be accurately specified. Am J Hum Genet 66(4):1310-27.
10. Hiekkalinna T, Schäffer AA, Lambert B, Norrgrann P, Göring HH, Terwilliger JD (2011) PSEUDOMARKER: a powerful program for joint linkage and/or linkage disequilibrium analysis on mixtures of singletons and related individuals Hum Hered 71(4):256-66.
11. Kruglyak L, Daly MJ, Lander ES (1995) Rapid multipoint linkage analysis of recessive traits in nuclear families, including homozygosity mapping. Am J Hum Genet 56: 519–527.
12. Lander ES, Green P (1987) Construction of multilocus genetic linkage maps in humans. Proc Natl Acad Sci USA 84: 2363–2367.
13. Lathrop GM, Lalouel J-M, Julier C, Ott J (1984) Strategies for multilocus linkage analysis in humans. Proc Natl Acad Sci USA 81:3443–3446.
14. Lathrop GM, Ott J (1990) Analysis of complex diseases under oligogenic models and intrafamilial heterogeneity by the LINKAGE programs. Am J Hum Genet Suppl 47:A188
15. Strauch et al. (2000) Parametric and Nonparametric Multipoint Linkage Analysis with
16. Imprinting and Two-Locus–Trait Models: Application to Mite Sensitization. Am J Hum Genet 66:1945–1957.

17. Thompson et al. (2003) A Full-Likelihood Method for the Evaluation of Causality of Sequence Variants from Family Data.
