# Supplementary material for: Digenic inheritance involving a muscle-specific protein kinase and the giant titin protein causes a skeletal muscle myopathy
Source: Nat Genet. 2024 Mar 1;56(3):395–407. doi: 10.1038/s41588-023-01651-0 (PMC10937387; doi:10.1038/s41588-023-01651-0)
Supplement: Supplementary file 2 — Reporting Summary [file 41588_2023_1651_MOESM2_ESM.pdf]

nature portfolio

Corresponding author(s): Volker Straub

Last updated by author(s): 10 October 2023

Reporting Summary

Nature Portfolio wishes to improve the reproducibility of the work that we publish. This form provides structure for consistency and transparency in reporting. For further information on Nature Portfolio policies, see our Editorial Policies and the Editorial Policy Checklist.

Statistics

For all statistical analyses, confirm that the following items are present in the figure legend, table legend, main text, or Methods section.

n/a	Confirmed	
☐	☒	The exact sample size (*n*) for each experimental group/condition, given as a discrete number and unit of measurement
☒	☐	A statement on whether measurements were taken from distinct samples or whether the same sample was measured repeatedly
☐	☒	The statistical test(s) used AND whether they are one- or two-sided<br>*Only common tests should be described solely by name; describe more complex techniques in the Methods section.*
☒	☐	A description of all covariates tested
☐	☒	A description of any assumptions or corrections, such as tests of normality and adjustment for multiple comparisons
☐	☒	A full description of the statistical parameters including central tendency (e.g. means) or other basic estimates (e.g. regression coefficient) AND variation (e.g. standard deviation) or associated estimates of uncertainty (e.g. confidence intervals)
☐	☒	For null hypothesis testing, the test statistic (e.g. *F*, *t*, *r*) with confidence intervals, effect sizes, degrees of freedom and *P* value noted<br>*Give P values as exact values whenever suitable.*
☒	☐	For Bayesian analysis, information on the choice of priors and Markov chain Monte Carlo settings
☒	☐	For hierarchical and complex designs, identification of the appropriate level for tests and full reporting of outcomes
☒	☐	Estimates of effect sizes (e.g. Cohen's *d*, Pearson's *r*), indicating how they were calculated

Our web collection on statistics for biologists contains articles on many of the points above.

Software and code

Policy information about availability of computer code

Data collection	Nikon A1R point scanning confocal microscope (Nikon, UK)<br>Nikon Elements AR, v5.21.03<br>Hitachi HT7800 120 kV TEM, using a EMSIS CMOS Xarosa high-resolution camera (Hitachi, Japan)<br>Radius software v2.0
Data analysis	For patients' RNA seq analysis:<br>featureCounts utility from the Subread package 2.0.0; calcNormFactors function from edgeR v3.28.1; AllelicImbalance67 software package v1.24.0<br>For zebrafish transcriptomics work:<br>FastQC v0.11.9 (https://www.bioinformatics.babraham.ac.uk/projects/fastqc/); STAR v2.7.3a; DESeq2 v1.28.1; topGO v2.38.1; ClueGO v2.5.9; Cytoscape 3.9.1; R v4.2.0 (R Core Team; https://www.r-project.org/); tidyverse package v1.3.1; rMATS v4.1.2<br>For the WGS mouse work:<br>BBmap suite v38.69; Varscan 2 v2.3.7; BCFTools v1.9; Variant Effect Predictor tool (Ensembl) https://www.ensembl.org/info/docs/tools/vep/index.html, https://www.ensembl.org/Tools/VEP<br>For variant analyses:<br>CADD v1.6 (https://cadd.gs.washington.edu/); YASARA v15.4.10; Mutation Mapper (https://www.cbioportal.org/mutation_mapper)<br>For statistical modelling:<br>LINKAGE package; MLINK; PSEUDOMARKER v2.0, (https://www.socscistatistics.com/tests/ztest/default2.aspx)

For manuscripts utilizing custom algorithms or software that are central to the research but not yet described in published literature, software must be made available to editors and reviewers. We strongly encourage code deposition in a community repository (e.g. GitHub). See the Nature Portfolio guidelines for submitting code & software for further information.

Data

Policy information about availability of data

All manuscripts must include a data availability statement. This statement should provide the following information, where applicable:

- Accession codes, unique identifiers, or web links for publicly available datasets
- A description of any restrictions on data availability
- For clinical datasets or third party data, please ensure that the statement adheres to our policy

Zebrafish RNA-seq data can be accessed in the ArrayExpress database at EMBL-EBI (www.ebi.ac.uk/arrayexpress) under accession number E-MTAB-12934. Mouse WGS data and human RNA-seq data can be accessed in the Sequence Read Archive (SRA) under accession number (PRJNA1027609 and PRJNA1027754, respectively). Control frequencies and variant information were extracted from gnomAD v2.1.1 (https://gnomad.broadinstitute.org). TTN variant information was obtained from Leiden Open Variation Database (https://databases.lovd.nl/shared/genes/TTN).

Human research participants

Policy information about studies involving human research participants and Sex and Gender in Research.

Reporting on sex and gender	We report on 31 males and two female patients. Gender is not relevant for the study.
Population characteristics	Patients with congenital myopathy and rare likely damaging variants in SRPK3 were included in the study. Agreggated and individual clinical data can be found in Supplementary Tables 1 and 2. The final cohort comprised 33 patients (29 males and 2 females). Age range was 1-77 years.
Recruitment	Recruitment was not performed for the study
Ethics oversight	All clinical information and biological material used in this collaborative study was collected after obtaining written informed consent from the patients or their legal guardians. Each sequencing study was approved by their relevant Health Research Authorities, as follows: Family A: Health Research Authority, NRES Committee East of England – Hatfield (REC 06/Q0406/33). Families B, W and X: Consent approved by the French legislation (Comité de Protection des Personnes Est IV DC-2012-1693). DNA storage and usage was IRB-approved: Comités de Protection des Personnes (CPP-Est DC-2012-1693). Family C: Ethics Committee of the National Center of Neurology and Psychiatry, Japan (A2011-081). Families D, R, V, Y and Z: NRES Committee North East – Newcastle & North Tyneside 1 (REC 08/H0906/28+5). Family E: EC approval (Number S52853) by Ethical Committee Research UZ / KU Leuven. Families H and U: by the Medical Review Ethics Committee, Region Arnhem–Nijmegen, Number 2011/188. Family L: NIH, National Institute of Neurological Disorders and Stroke (NINDS), Institutional Review Board (Protocol 12-N-0095). Family M: Ethics committee of the Helsingin ja Uudenmaan sairaanhoitopiiri (HUS, statement number 195/13/03/00/11). Family N: University of Pretoria Faculty of Health Sciences Research Ethics Committee Ref 296/2019. Families O and P: Boston Children's Hospital Institutional Review Board (Protocol #03-08-128R). Family Q: Rare Genomes Project (Protocol #: 2016P001422) study, approved by the IRB at Massachusetts General Brigham. Family S: New Zealand Health and Disability Ethics Committee - approval number 20/NTB/139. Families F, G, J, K and T: tested through their respective national diagnostic health services.

Note that full information on the approval of the study protocol must also be provided in the manuscript.

Field-specific reporting

Please select the one below that is the best fit for your research. If you are not sure, read the appropriate sections before making your selection.

☒ Life sciences ☐ Behavioural & social sciences ☐ Ecological, evolutionary & environmental sciences

For a reference copy of the document with all sections, see nature.com/documents/nr-reporting-summary-flat.pdf

Life sciences study design

All studies must disclose on these points even when the disclosure is negative.

Sample size	Statistical methods were not used to determine sample size. The study cohort consisted of 33 congenital myopathy patients with rare variants in SRPK3. Disease cohorts consisted of 170, 94 and 56 individuals with confirmed diagnosis of CAPN3-, DYSF-, and ANO5-related muscular dystrophy, respectively, identified through previous sequencing projects.
Data exclusions	No data was excluded.
Replication	Zebrafish single myofiber staining images were taken from >15 pooled fish per genotype. Zebrafish EM images were taken from 3-5 pooled fish per genotype. Zebrafish transcriptomics was performed with n=6 for each condition. In vitro phosphorylation assay was performed in quadruplicate. TTN western blots were repeated twice, from the same muscle lysates. All attempts were successful.

Randomization	The study was not randomised.
Blinding	Investigators were blinded to sex and affected status of family members during segregation analysis.

Reporting for specific materials, systems and methods

We require information from authors about some types of materials, experimental systems and methods used in many studies. Here, indicate whether each material, system or method listed is relevant to your study. If you are not sure if a list item applies to your research, read the appropriate section before selecting a response.

Materials & experimental systems

n/a	Involved in the study	
☐	☒	Antibodies
☐	☒	Eukaryotic cell lines
☒	☐	Palaeontology and archaeology
☐	☒	Animals and other organisms
☒	☐	Clinical data
☒	☐	Dual use research of concern

Methods

n/a	Involved in the study	
☒	☐	ChIP-seq
☒	☐	Flow cytometry
☒	☐	MRI-based neuroimaging

Antibodies

Antibodies used	Alexa Fluor® 488 Phalloidin Invitrogen A12379 (1:80)<br>mouse monoclonal anti α-actinin Sigma A7811 (1:200)<br>rabbit polyclonal anti-sarcomeric α Actinin Cell Signaling Technologies #3134, Lot #2 (1:25)<br>mouse monoclonal anti Titin Merck T9030, clone T11, ascites fluid, lot #029M4835V (1:200)<br>goat anti-mouse Alexa Fluor® 488 secondary antibody ThermoFisher A11001 (1:500)<br>goat anti-rabbit Alexa Fluor® 594 secondary antibodies ThermoFisher A11037 (1:250)<br>V5 tag ThermoFisher R960-25, SV5-Pk1 (1:5000)<br>GFP ThermoFisher A11122 (1:5000)<br>rabbit polyclonal anti-titin M10 (M10-1) as described in Hackman et al. Neuromuscul Disord 18:922–928 (2008)(1:300)<br>mouse monoclonal anti-titin M10 (11-4-3) as described in Evilä et al. Ann Neurol 75:230–240 (2014) (1:150)<br>rabbit polyclonal anti-titin Z1Z2 Myomedix, TTN-1 (1:1500)<br>mouse monoclonal anti-titin (distal I-band) Enzo Life Sciences, ALX-BC-3010-S, F146.9B9 (1:1000)
Validation	Hackman et al. Neuromuscul Disord 18:922–928 (2008)<br>Evilä et al. Ann Neurol 75:230–240 (2014)

Eukaryotic cell lines

Policy information about cell lines and Sex and Gender in Research

Cell line source(s)	293T cells (ATCC, CRL-3216)
Authentication	Cells were commercially available and were not authenticated.
Mycoplasma contamination	Cell line tested negative for mycoplasma contamination
Commonly misidentified lines (See ICLAC register)	None used

Animals and other research organisms

Policy information about studies involving animals; ARRIVE guidelines recommended for reporting animal research, and Sex and Gender in Research

Laboratory animals	Adult zebrafish (Danio erio) mutant lines srpk3(sa18907) and ttn.1(sa5562) were used to obtain all genotype combinations. Double mutants larvae were analysed at 5dpf.
Wild animals	No wild animals were used in this study
Reporting on sex	This information has not been collected
Field-collected samples	No field-collected samples were used in this study
Ethics oversight	Zebrafish were maintained in accordance with UK Home Office regulations, UK Animals (Scientific Procedures) Act 1986, under

Ethics oversight	project licences 70/7606 and P597E5E82. All animal work was reviewed by The Wellcome Trust Sanger Institute Ethical Review Committee.

Note that full information on the approval of the study protocol must also be provided in the manuscript.
