# Supplementary material for: Digenic inheritance involving a muscle-specific protein kinase and the giant titin protein causes a skeletal muscle myopathy
Source: Nat Genet. 2024 Mar 1;56(3):395–407. doi: 10.1038/s41588-023-01651-0 (PMC10937387; doi:10.1038/s41588-023-01651-0)
Supplement: Supplementary file 6 — Editorial assessment report. [file 41588_2023_1651_MOESM6_ESM.pdf]

Contents of this report

- **Manuscript details**: overview of your manuscript and the editorial team.
- **Review synthesis**: summary of the reviewer reports provided by the editors.
- **Editorial recommendation**: personalized evaluation and recommendation from all 3 journals.
- **Annotated reviewer comments**: the referee reports with comments from the editors.
- **Open research evaluation**: advice for adhering to best reproducibility practices.

About the editorial process

Because you selected the **Nature Portfolio Guided Open Access option**, your manuscript was assessed for suitability in three of our titles publishing high-quality work across the spectrum of genetics research: *Nature Genetics, Nature Communications*, and *Communications Biology*. More information about Guided Open Access can be found here.

Collaborative editorial assessment

Your editorial team discussed the manuscript to determine its suitability for the Nature Portfolio Guided OA pilot. Our assessment of your manuscript takes into account several factors, including whether the work meets the **technical standard** of the Nature Portfolio and whether the findings are of **immediate significance** to the readership of at least one of the participating journals in the Nature Portfolio Guided Open Access genetics cluster.

Peer review

Experts were asked to evaluate the following aspects of your manuscript:

- **Novelty** in comparison to prior publications;
- **Likely audience** of researchers in terms of broad fields of study and size;
- **Potential impact** of the study on the immediate or wider research field;
- **Evidence** for the claims and whether additional experiments or analyses could feasibly strengthen the evidence;
- **Methodological detail** and whether the manuscript is reproducible as written;
- Appropriateness of the literature review.

Editorial evaluation of reviews

Your editorial team discussed the potential suitability of your manuscript for each of the participating journals. They then discussed the revisions necessary in order for the work to be published, keeping each journal's specific editorial criteria in mind.

Journals in the Nature portfolio will support authors wishing to transfer their reviews and (where reviewers agree) the reviewers' identities to journals outside of Springer Nature.

If you have any questions about review portability, please contact our editorial office at guidedoa@nature.com.

Manuscript details

Tracking number	Submission date	Decision date
GUIDEDOA-21-00084	29 March 2021	14 July 2021

**Title**	Digenic inheritance involving a muscle specific protein kinase and the giant titin protein causes a skeletal muscle myopathy	**Corresponding author**	Ana Topf<br>**Affiliation:** John Walton Muscular Dystrophy Research Centre
**Preprint information**	There is a preprint of this manuscript posted here.	**Peer review type**	Single-blind

Editorial assessment team

**Primary editor**	Kyle Vogan<br>**Home Journal:** *Nature Genetics*, ORCID: 0000-0001-9565-9665<br>**Email:** k.vogan@us.nature.com
**Editorial team members**	**Ingrid Knarston**, *Nature Communications*, ORCID: 0000-0002-0932-8649<br>**George Inglis**, *Communications Biology*, ORCID: 0000-0002-9069-5242
**About your primary editor**	Kyle obtained his Ph.D. from McGill University under the supervision of Philippe Gros, using the classical mouse mutants Splotch and Loop-tail to study the genetics of neural tube closure. For his postdoctoral work, he joined Cliff Tabin at Harvard Medical School where he studied the developmental mechanisms underlying vertebrate left-right axis specification. He joined the journal in 2003.

Editorial assessment and review synthesis

**Editor's summary and assessment**	The authors present evidence that deleterious variants in *SRPK3* (an X-linked gene encoding a serine/arginine protein kinase) lead to a progressive childhood-onset skeletal muscle myopathy only when in combination with heterozygous variants in *TTN*. In accordance with the findings in their myopathy patients, they observed that the loss of one ttn.1 wild-type allele in srpk3-null zebrafish resulted in severe muscle pathology. *SRPK3* was previously known from mouse knockout studies to be essential for muscle growth and homeostasis (refs. 13, 14, 16), with phenotypes reminiscent of a human centronuclear myopathy, so the main point of novelty and interest is that this represents a rare example of digenic inheritance for human disease.
**Editorial synthesis of reviews**	The reviewers consider the human genetic findings convincing and of interest to a broad audience. Their main criticisms and suggestions for improvement are focused on the molecular and phenotypic analyses of the human patient biopsies and zebrafish mutants.<br><br>When revising the paper for *Nature Genetics*, we ask that you extend these aspects of the study along the lines requested by the reviewers (to the extent that is feasible based on available biopsy material) and revise the text and display items for clarity throughout taking into account their specific queries. Although we agree it would be interesting to overexpress the mouse Titin missense variants in zebrafish and assess potential phenotypes, we would not require this experiment as a condition for further consideration.<br><br>When revising the paper for *Nature Communications*, we would ask that you extend analyses on mutation impact, specifically how these mutations impact RNA- and protein-level changes in zebrafish and human (pending muscle biopsy availability). We would like you to provide improved quantification/quality of Western blots and extend characterization of zebrafish mutants, including antibody staining and motility functional testing. We would not require the work on overexpression of the mouse Titin variants in zebrafish or functional testing of zebrafish mutants for cardiac function.<br><br>When revising the paper for *Communications Biology*, we would ask that you improve the quality of staining and Western blots for proteins of interest and to make all textual revisions suggested by reviewers, but would not require additional functional work in zebrafish models.

Editorial recommendation

nature genetics — Major Revisions

We think the evidence for digenic inheritance of this human myopathy will be of interest to our readership, but we think the mechanistic aspects of the study should be strengthened by performing more detailed molecular and phenotypic characterization of available patient biopsies and zebrafish mutants.

nature communications — Major Revisions

We think this study would be of high interest to our readership. We would like to see further validation of RNA- and protein-level effects of reported variants and further phenotypic characterisation of the zebrafish model to strengthen this study.

communications biology — Minor Revisions

We believe this is a nicely-designed and -controlled study that could be strengthened with minor experiments, including performing additional Western blots for proteins of interest, staining for relevant markers, and textual revisions addressing limitations or clarifying points of discussion (as best outlined by Reviewer #2).

Next steps

Recommendation Summary

- **Option 1:** Revise for consideration at *Nature Genetics*
- **Option 2:** Revise for consideration at *Nature Communications*
- **Option 3:** Revise for consideration at *Communications Biology*

See the previous page for details

Revision

If you would like to follow our recommendation, please upload the revised manuscript, along with your point-by-point response to the reviewers' reports and editorial advice **using the link provided in the decision letter**.

Revision checklist

- Cover letter, stating to which journal you are submitting
- Revised manuscript
- Point-by-point response to reviews
- Updated **Reporting Summary** and **Editorial Policy Checklist**
- Supplementary materials (if applicable)

Submission elsewhere

Within the Nature Portfolio

Springer Nature provides authors with the ability to transfer a manuscript within the Nature Portfolio, without the author having to upload the manuscript data again. To use this service, please **follow the transfer link provided in the decision letter**.

Note that any decision to opt in to *In Review* at the original journal is not sent to the receiving journal on transfer. You can opt in to *In Review* at receiving journals that support this service by choosing to modify your manuscript on transfer.

To a journal outside of Nature Portfolio

We can share the reviews with another journal outside of the Nature Portfolio if requested. You will need to request that the receiving journal office contacts us at guidedOA@nature.com. We have included editorial guidance below in the reviewer reports and open research evaluation to aid in revising the manuscript for publication elsewhere.

Annotated reviewer reports

The editors have included some additional comments on specific points raised by the reviewers below, to clarify requirements for publication in the recommended journal(s). However, please note that all points should be addressed in a revision, even if an editor has not specifically commented on them.

Reviewer #1

**Reviewer #1**	This reviewer has not chosen to waive anonymity. The reviewer's identity can only be shared with representatives of an established journal editorial office.
**Reviewer #1 expertise**<br>Summarised by the editor	This reviewer has expertise in skeletal muscle diseases and zebrafish models.
**Editor's comments about this review**	Reviewer #1 considers the human genetic evidence for digenic inheritance very interesting but thinks several experiments could be added to strengthen the study's claims and provide further mechanistic insights. We note that Reviewer #3 has raised similar concerns, and we agree that improving the quality and depth of the mechanistic aspects of the study would be required for publication in *Nature Genetics*.<br><br>For consideration at *Nature Communications*, we would like to see further validation of mutation impacts and phenotypic characterization as outlined below, but would consider overruling Reviewer #1's request for further study on zebrafish mutant cardiac function.<br><br>For consideration at *Communications Biology*, we would ask that you improve the quality of Western blots, acknowledge the limited availability of data from zebrafish models in the Discussion, and (if feasible), perform additional staining in zebrafish muscle.

Reviewer #1 comments

**Overview**	Topf et al present a novel myopathy caused by the digenic inheritance of variants in Srpk3 and Ttn. They present numerous families that support this association, as well as data from other diseases to show that co-inheritance is not by chance (i.e. truncating Ttn variants are not found in these diseases). In addition, they provide support from zebrafish mutants, where srpk3 -/- fish only have a phenotype in the setting of ttn +/- or -/-. Overall, this is an extremely compelling article presenting one of the few examples of digenic inheritance as the cause of a Mendelian

	appearing disease. There are some areas where additional data would strengthen the proof of this association and provide insight into disease mechanism.<br>This study has high potential impact, as digenic inheritance may be a potential explanation for many currently unsolved cases of rare disease.<br>There are areas where data can be obtained or added that would strengthen the claims of the manuscript:

Specific comments

#	Reviewer comment	Editorial comment
1	The authors appear to have access to several biopsies from the patients (and potentially unaffecteds as well?). Given the proposed function(s) of Srpk3, it would be informative to understand how the presence of both mutations impacts transcriptional changes in the muscle. This would be most informative related to Ttn (are Ttn transcripts altered by Srpk3 mutation?), but also to see if Ttn variation alters Srpk3 dependent RNA regulation.	For consideration at *Nature Genetics* and *Nature Communications*, please extend the transcriptome analyses in the patient biopsies as requested.
2	Also from the biopsies, the study looks at TTN levels. The Westerns are not the most compelling data, as it does look like there might be some expression in at least one of the digenic patients. Is there an opportunity to more precisely define TTN quantification and post translational modification? Perhaps by using IP/MS and direct interrogation of TTN?	For consideration at *Nature Genetics* and *Nature Communications*, please improve the Western blots or use alternate approaches to quantify TTN protein levels. While *Communications Biology* would also require higher-quality Western blots, it would not be necessary to utilize a secondary quantification method.
3	The zebrafish data is quite sparse, making it hard to contextualize the model and understand whether it provides validation of the human data. As presented it only minimally adds to the proof.	
4	No information is provided about the impact of the mutation on Srpk3 RNA and protein. Presumably this is a KO but it would be important to investigate at minimum with RNA analysis.	It would be important to evaluate whether the mutation impacts *Srpk3* RNA or protein levels.
5	Only phalloidin staining is provided as phenotypic	For consideration at *Nature*

	characterization. This is pretty minimal in terms of a data set. In terms of histopathology, what about proteins that illuminate other muscle structures? There are good antibodies that work in zebrafish that mark the thin filament, the Z band, and the triad (as well as the DAPC). These can be used in whole mount or on myofibers, and would provide more of a picture of the impact of the double mutation, and also enable an opportunity to better compare with the human pathology. Similarly, it would be important to show EM from the zebrafish to see whether there are analogous structural changes.	*Genetics* and *Nature Communications*, please provide a more detailed characterization of the muscle pathology in the zebrafish mutants. While *Communications Biology* would encourage you to further examine the histopathology of zebrafish mutants, it would not be necessary to use EM.
**6**	No functional data is provided from the zebrafish. Is swim behavior impaired? How about survival? Also, what about cardiac function (heart rhythm and ventricular shortening are relatively straight forward to enumerate).	This point would not be necessary for consideration at *Nature Communications* or *Communications Biology*.
**7**	What are TTN levels (RNA and protein) in the srpk3 -/-, ttn +/-?	Similar to Point #4 from this reviewer, we would encourage you to assess *TTN* RNA and/or protein levels in these models.
**8**	Are there alterations in the global transcriptome in srpk3 -/-? Are they impacted by the presence of a heterozygous ttn variant? How do they compare to the human biopsies?	This point would not be necessary for consideration at *Communications Biology*.
**9**	The data in general appears valid and reproducible. The Western blots of TTN are somewhat difficult to interpret and also lack quantification. The caveat is that these are extremely technically challenging. An alternative way (such as IP/MS) at looking at TTN levels might be helpful in this regard, as this is a pivotal point for the study conclusions.	While *Communications Biology* would not require IP or MS to be utilized to look at TTN levels, we would ask that you quantify these blots and consider using separate representative images.

Reviewer #2	
**Reviewer #2**	This reviewer has not chosen to waive anonymity. The reviewer's identity can only be shared with representatives of an established journal editorial office.
**Reviewer #2 expertise**<br>Summarised by the editor	This reviewer has expertise in human genetics, rare diseases, and X-linked disorders.
**Editor's comments about this review**	Reviewer #2 finds the human genetic evidence for digenic inheritance interesting and convincing. This reviewer notes specific aspects of the human genetic data that should be revised for clarity. The reviewer's comments should be addressed with textual revisions for consideration at *Nature Genetics*, *Nature Communications*, or *Communications Biology*.
**Reviewer #2 comments**	
**Overview**	I have read the manuscript by Töpf and colleagues on "Digenic inheritance involving a muscle specific protein kinase and the giant titin protein causes a skeletal muscle myopathy" with great interest. The authors report that deleterious variants in the X-linked SRPK3 gene lead to a progressive childhood-onset skeletal muscle myopathy only when in combination with heterozygous TTN variants. Double mutant zebrafish recapitulated the clinical phenotype.<br>The results are original and significant, will be of interest to others in the community, and the manuscript is well written. This Reviewer thinks that this is a significant contribution to the field. The work is very convincing.

Specific comments

#	Reviewer comment	Editorial comment
1	**Abstract:** Are the deleterious variants all loss-of function variants? If so, I suggest adding this information.	
2	Please use "variant" or "pathogenic variant" instead of "mutation" throughout the manuscript.	
3	**Line 172:** *"Segregation analyses (in 16 families) showed that SRPK3 variants were always inherited from an*	

	unaffected mother, with none being de novo."<br>Would it be possible to test if the variants were de novo in the unaffected mothers?	
**4**	How did the authors define "partially skewed X-inactivation"?	Please clarify your interpretation of the X-inactivation data.
**5**	**Line 199:** The authors pointed out that *"Interestingly, individual VII:5, a female SRPK3 carrier showing a pronounced skewed X-inactivation pattern (3:97) but no TTN variant, was clinically unaffected."*<br>This Reviewer wonders why this is interesting.	
**6**	**Line 201:** *"Also unaffected was individual NI:2,……"*<br>Please revise. This Reviewer understood that all females with co-segregating SRPK3 and TTN variants, except for the two carriers in family U are unaffected.	
**7**	Results on the genetic background of the *Srpk3* KO mouse are interesting, but descriptive and it remains unclear whether the *Ttn* variants contribute to the observed phenotype.	It would be important to acknowledge this limitation if revising for *Communications Biology*.
**8**	**Line 289:** *"Six females also carried both…".*<br>Fig. 1C shows nine females with both SRPK3 and TTN variants.	
**9**	While numbers are still small, X-inactivation results are repeated rather instead of being discussed.	
**10**	Do the authors have any evidence that SRPK3 and titin are complex partners?	
**11**	**Extended Data Fig.2:** p.Glu455Ly is not a SRPK3 variant of Extended Table 1	
**12**	Again, numbers are still small. Nevertheless, this Reviewer wonders whether it would make sense mentioning that all *SRPK3* missense variants identified so far are located in the kinase domains.	

Reviewer #3

**Reviewer #3**	This reviewer has not chosen to waive anonymity. The reviewer's identity can only be shared with representatives of an established journal editorial office.
**Reviewer #3 expertise**<br>Summarised by the editor	This reviewer has expertise in cardiac diseases, muscle, and zebrafish models.
**Editor's comments about this review**	Reviewer #3 thinks the findings are very interesting but expresses concerns about the quality of the presentation and requests additional data to support the study's claims. We note that Reviewer #1 has raised similar concerns, and we agree that improving the quality and depth of the mechanistic aspects of the study would be required for publication in *Nature Genetics*.<br><br>For consideration at *Nature Communications*, we would like to see expanded phenotypic characterization of zebrafish mutants and improved clarity of data presented, as outlined below. We would overrule Reviewer #3's request for further work on overexpression of mouse Titin in zebrafish.<br><br>For consideration at *Communications Biology*, we would simply ask that you remove the statement about NMD or use qPCR to investigate reduced mRNA levels, improve Western blot quality, and examine SRPK3 and RBM20 protein levels. If feasible, we would also ask that you perform additional staining for sarcomeric markers.

Reviewer #3 comments

**Overview**	In this manuscript the authors present an example of digenic inheritance related to a potential interaction between SRPK3, a X-linked kinase, and TTN, the giant muscle protein. They analyzed a cohort of patients with deleterious variants in *SRPK3* in combination with heterozygous variants in *TTN*. To support their hypothesis of digenic inheritance they used zebrafish as animal model by creating double carrier fish of the *srpk3* and *ttn.1* variants.<br><br>This new finding is very interesting, but the data are not very well presented, therefore I have a number of major concerns.

Specific comments

#	Reviewer comment	Editorial comment
1	**Line 165:** The authors write about NMD (nonsense	This should be a straightforward

	mediated mRNA decay). Is this a hypothesis? In the Extended Fig. 1 there is no proof related to it. A qPCR would be nice to prove the downregulation of mRNA levels.	and informative experiment to add, for consideration at *Nature Genetics* or *Nature Communications*. For *Communications Biology*, we would ask that you either remove the statement about NMD or perform this qPCR experiment.
**2**	**Line 215:** The authors describe for the patient NII:1 elevated levels of CK. Why do the authors report this fact? Could this influence the phenotype? Was this condition in the patient related only to the specific moment/period the patient was tested or is this a standard condition of the patient?	
**3**	**Lines 216-224:** the authors describe too roughly the Fig 2A and without having a look on the figure legend it is impossible to understand to which patient the description relates. It would be nice if the authors briefly mention in the text whether the finding occurs in all the patients or only in few. Please rephrase this part.	Please clarify the presentation of these findings.
**4**	**Line 231:** Based on which criteria the authors decided to perform the Western blot using only those 4 patients? And why didn't they consider to perform the Western blot analysis also using samples from patients with a myopathic phenotype (families M and V)?	
**5**	**Fig. 2C:** The quality of the WB is not adequate. Please provide better quality pictures? Related to the N-term titin blot, the authors describe the presence of a clear titin band in sample DI:1. Actually the band is not really clear and there are a lot of smears for the N-term and I-band blots.<br><br>The authors should consider to perform a new Western blot decreasing the percentage of the gel (to 1.8-2.5%) for a better quality (as published from Swist et al. 2020 doi: 10.1038/s41467-020-18131-2).	Reviewer #1 raised a similar concern. Please improve the quality of the Western blot and/or use an alternate method to quantify TTN protein levels, for consideration at *Nature Genetics* and *Nature Communications*. *Communications Biology* would only require higher-quality Western blots.
**6**	The authors performed the staining in zebrafish embryos at 5days post fertilization. It would be nice to know for how long the embryos that display the muscle fiber disruption survive. Do they survive to adulthood? Is	Reviewer #1 raised similar concerns. Please provide more a detailed characterization of the muscle pathology in the zebrafish

	motility affected in these embryos? Is sarcomerogenesis affected early on (24hpf) or is this degeneration of muscle fibres over time (e.g. day2-5)? Is heart function also affected in the double mutation carriers? The description of muscle fibres disruption is superficial and should be expanded by time-course analyses, by the use of more sarcomeric markers and electron microscopy imaging.	model for consideration at *Nature Genetics* or *Nature Communications*.<br><br>*Communications Biology* would ask that you do additional staining, but no additional experiments about motility or sarcomerogenesis would be necessary.
**7**	**Lines 277-284:** *srpk3* KO mice show a muscle phenotype and it is not known if ttn variants are contributing to the phenotype. On the other hand, the *srpk3* mutant zebrafish line does not show any muscle phenotypes. Hence, it would be interesting to overexpress the specific titin missense variants (detected in mice) in zebrafish (by Tol2 transgenesis) and to analyze a potential phenotype.	While we think this would be a good experiment, we would not require it as a condition for further consideration at *Nature Genetics*, *Nature Communications*, or *Communications Biology*.
**8**	**Lines 322-329:** It would be nice if the authors could show SRPK3 and RBM20 protein levels by Western blotting (using the same samples as used for Fig. 2C). Is RBM20 mislocalizing (immunostainings)?	The editors at *Nature Genetics* and *Nature Communications* encourage you to perform these additional experiments.

Open research evaluation

Data availability

Data availability statement

Thank you for including a Data Availability statement. However, we noted that you have only indicated that data are available upon request. The data availability statement must make the conditions of access to the “minimum dataset” that are necessary to interpret, verify and extend the research in the article, transparent to readers.

In addition, Nature Portfolio policies include a strong preference for research data to be archived in public repositories. For data types without specific repositories, we recommend that data are deposited in a generalist repository such as figshare or Dryad. More information about our data availability policy can be found here: https://www.nature.com/nature-portfolio/editorial-policies/reporting-standards#availability-of-data

See here for more information about formatting your Data Availability Statement: http://www.springernature.com/gp/authors/research-data-policy/data-availability-statements/12330880

Mandatory data deposition

For DNA and RNA sequencing data, submission to a community-endorsed, public repository is mandatory for publication in a Nature Portfolio journal (unless data sharing is restricted due to patient consent or privacy laws) and is best practice for publication in any venue. Accession numbers must be provided in the paper. Examples of appropriate public repositories are listed below:

- GenBank
- Sequence Read Archive (WGS or WES data)
- The European Nucleotide Archive (ENA)

For more information on mandatory data deposition policies at the Nature Portfolio, please visit http://www.nature.com/authors/policies/availability.html#data

For an up-to-date list of approved repositories for each mandatory data type, please visit https://www.springernature.com/gp/authors/research-data-policy/repositories/12327124

Other data requests

We strongly encourage the deposition of your full microscopy image data sets in the Image Data Resource: https://idr.openmicroscopy.org/about

For an up-to-date list of approved repositories for each mandatory data type, please visit https://www.springernature.com/gp/authors/research-data-policy/repositories/12327124

All source data underlying the graphs and charts presented in the main figures must be made available as Source Data (in Excel or text format) or via a generalist repository (e.g., Figshare or Dryad). This is mandatory for publication in a Nature Portfolio journal, but is also best practice for publication in any venue.

The following figures require associated source data: Extended Data Figure 3

Our journals strongly supports public availability of data and custom code associated with the paper in a persistent repository where they can be freely and enduringly accessed or as a supplementary data file when no appropriate repository is available. If data and code can only be shared on request, please explain why in your data Availability Statement, and also in the correspondence with your editor. For more information, please refer to https://www.nature.com/nature-research/editorial-policies/reporting-standards#availability-of-data

Please ensure that datasets deposited in public repositories are now publicly accessible, and that accession codes or DOI are provided in the "Data Availability" section. As long as these datasets are not public, we cannot proceed with the acceptance of your paper. For data that have been obtained from publicly available sources, please provide a URL and the specific data product name in the data availability statement. Data with a DOI should be further cited in the methods reference section.

Please also supply uncropped and unprocessed scans of the most important blots in the Source Data file or as a supplementary figure in the Supplementary Information. This should be cited once in the Methods section. For an example of presentation of full scan blots, see the Source Data file of https://www.nature.com/articles/s41467-020-16984-1#Sec35 and for more information, please refer to https://www.nature.com/nature-research/editorial-policies/image-integrity

Please note that full scans are missing for Figure 2c and Extended Data Figures 1a, b.

Data citation

Please cite (within the main reference list) any datasets stored in external repositories that are mentioned within their manuscript. For previously published datasets, we ask that you cite both the related research article(s) and the datasets themselves. For more information on how to cite datasets in submitted manuscripts, please see our data availability statements and data citations

policy: https://www.nature.com/documents/nr-data-availability-statements-data-citations.pdf

Citing and referencing data in publications supports reproducible research, by increasing the transparency and provenance tracking of data generated or analysed during research. Citing data formally in reference lists also helps facilitate the tracking of data reuse and may help assign credit for individuals' contributions to research. A number of Springer Nature imprints are signatories of the Joint Declaration on Data Citation Principles, which stress the importance of data resources in scientific communication.

Ethics

Because your study includes human participants, confirmation that all relevant ethical regulations were followed is needed, and that informed consent was obtained. This must be stated in the Methods section, including the name of the board and institution that approved the study protocol.

Because your study uses live vertebrates, a statement affirming that you have complied with all relevant ethical regulations for animal testing and research is necessary. A statement explicitly confirming if the study received ethical approval, including the name of the board and institution that approved the study protocol is also required. The species, strain, sex and age of animals should be included.

Reporting and reproducibility

Reporting

Please include the full, uncropped blot/gel images as a Source Data file and cite this Source Data file in the corresponding figure legend.

Reproducibility

Please state in the legends how many times each experiment was repeated independently with similar results. This is needed for all experiments, but is particularly important wherever results from representative experiments (such as micrographs) are shown. If space in the legends is limiting, this information can be included in a section titled "Statistics and Reproducibility" in the methods section.

Please note that this information is missing in the legends of **Figures 2a (a-l), c; 3a-f and Extended Data Figures 1a, b.**

Statistics

Wherever statistics have been derived (e.g. error bars, box plots, statistical significance) the legend needs to provide and define the n number (i.e. the sample size used to derive statistics) as a precise value (not a range), using the wording "n=X biologically independent samples/animals/cells/independent experiments/n= X cells examined over Y independent experiments" etc. as applicable.

Statistics such as error bars, significance and p values cannot be derived from n<3 and must be removed from all such cases.

We strongly discourage deriving statistics from technical replicates, unless there is a clear scientific justification for why providing this information is important. Conflating technical and biological variability, e.g., by pooling technically replicates samples across independent experiments is strongly discouraged. (For examples of expected description of statistics in figure legends, please see the following https://www.nature.com/articles/s41467-019-11636-5 or https://www.nature.com/articles/s41467-019-11510-4).

All error bars need to be defined in the legends (e.g. SD, SEM) together with a measure of centre (e.g. mean, median). For example, the legends should state something along the lines of "Data are presented as mean values +/- SEM" as appropriate.

All box plots need to be defined in the legends in terms of minima, maxima, centre, bounds of box and whiskers and percentile.

The figure legends must indicate the statistical test used. Where appropriate, please indicate in the figure legends whether the statistical tests were one-sided or two-sided and whether adjustments were made for multiple comparisons.

For null hypothesis testing, please indicate the test statistic (e.g. F, t, r) with confidence intervals, effect sizes, degrees of freedom and P values noted.

Please provide the test results (e.g. P values) as exact values whenever possible and with confidence intervals noted.

Legends requiring revision:

1. Please indicate the statistical test used for data analysis and where appropriate, please specify whether it was one-sided or two-sided and whether adjustments were made for multiple comparisons, in the legend of **Extended Data Figure 3**.
2. Please note that the exact p value should be provided, when possible, in the legend of **Extended Data Figure 3**.

Data presentation

Please ensure that data presented in a plot, chart or other visual representation format shows data distribution clearly (e.g. dot plots, box-and-whisker plots). When using bar charts, please overlay the corresponding data points (as dot plots) whenever possible and always for n ≤ 10. (Please see the following editorial for the rationale behind this request and an example https://www.nature.com/articles/s41551-017-0079).

Quantitative comparisons between samples on different gels/blots are discouraged; if this is unavoidable, the figure legend must state that the samples derive from the same experiment and that gels/blots were processed in parallel.

- Vertically sliced images that juxtapose lanes that were non-adjacent in the gel must have a clear separation or a black line delineating the boundary between the gels. Loading controls (e.g. GAPDH, actin) must be run on the same blot.
- Sample processing controls run on different gels must be identified as such in the figure legends, and distinctly from loading controls.
- All blots and gels must be accompanied by the locations of molecular weight/size markers. Blots should be cropped such that at least one marker position is present.

Please ensure that all micrographs include a scale bar and this scale bar is defined on the panels or in the figure legends.

1. Please note that scale bar is missing for **Figures 3a-f.**
2. Please note that the scale bar needs to be defined for **Figures 2a (a-l).**

Other notes

We have included as an attachment to the decision letter a version of your Reporting Summary with a few notes. This is mainly for your information, but we hope it is helpful when preparing your revised manuscript. If you decide to resubmit the manuscript for further consideration, please be sure to include an updated Reporting Summary.
