# Supplementary material for: Digenic inheritance involving a muscle-specific protein kinase and the giant titin protein causes a skeletal muscle myopathy
Source: Nat Genet. 2024 Mar 1;56(3):395–407. doi: 10.1038/s41588-023-01651-0 (PMC10937387; doi:10.1038/s41588-023-01651-0)
Supplement: Supplementary file 7 — Unprocessed western blots. [file 41588_2023_1651_MOESM7_ESM.pdf]

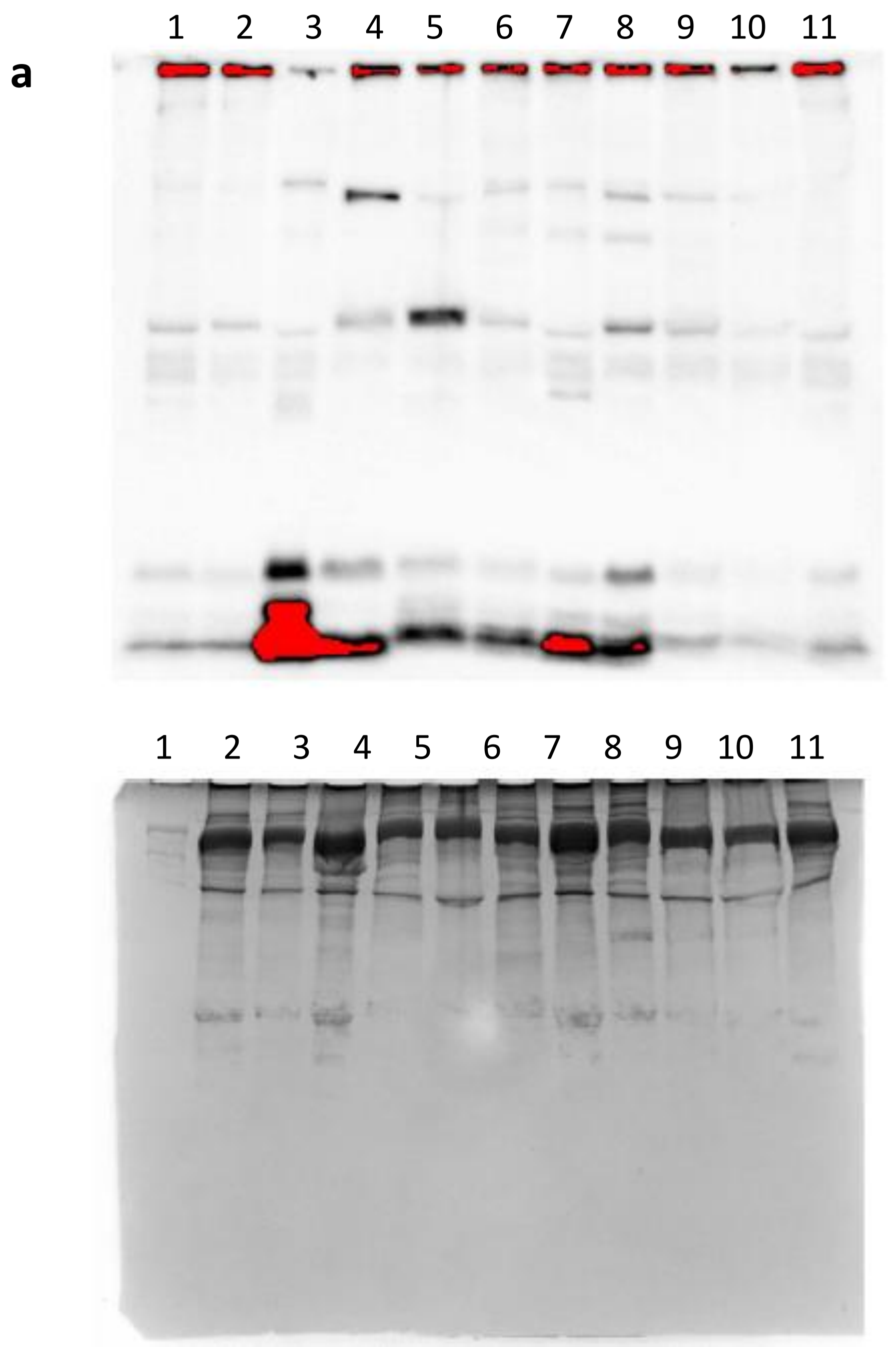

1: Control
2: Control
3: not included
4: LII:1
5: Control
6: YII:3
7: DI:1
8: DII:1
9: XII:3
10: XIII:1
11: control

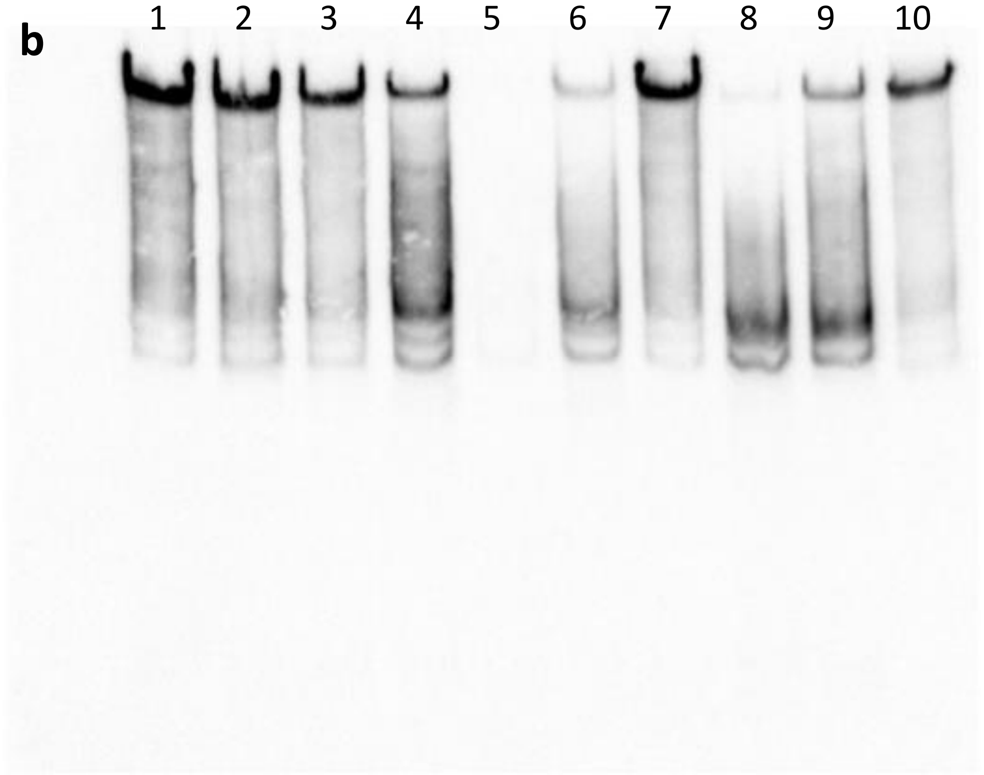

1: Control
2: Control
3: disease control
4: DI:1
5: not included
6: XIII:1
7: control
8: XII:3
9: YII:3
10: control

c

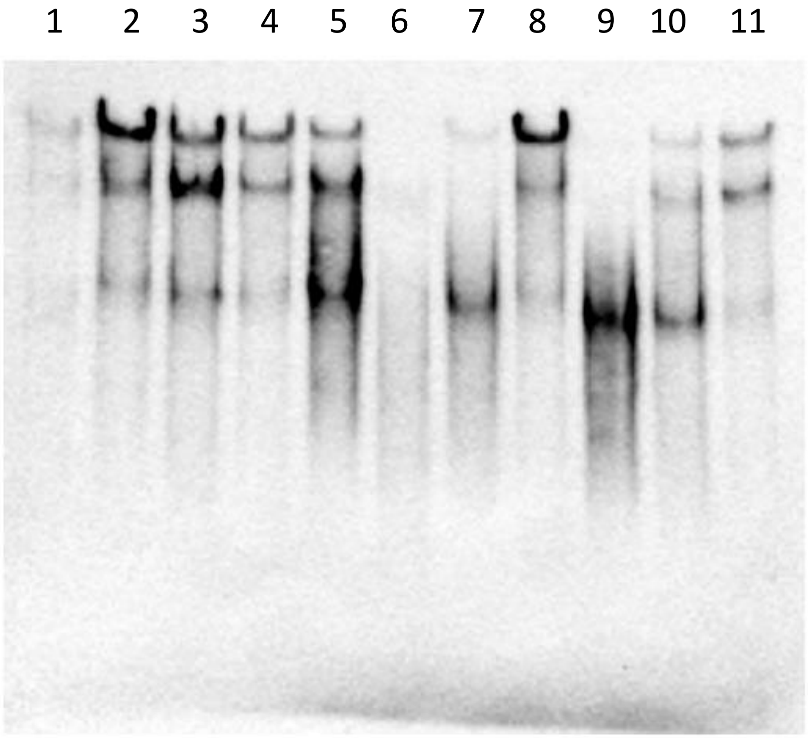

1: not included
2: Control
3: Control
4: Disease control
5: DI:1
6: not included
7: XIII:1
8: control
9: XII:3
10: YII:3
11: control

d

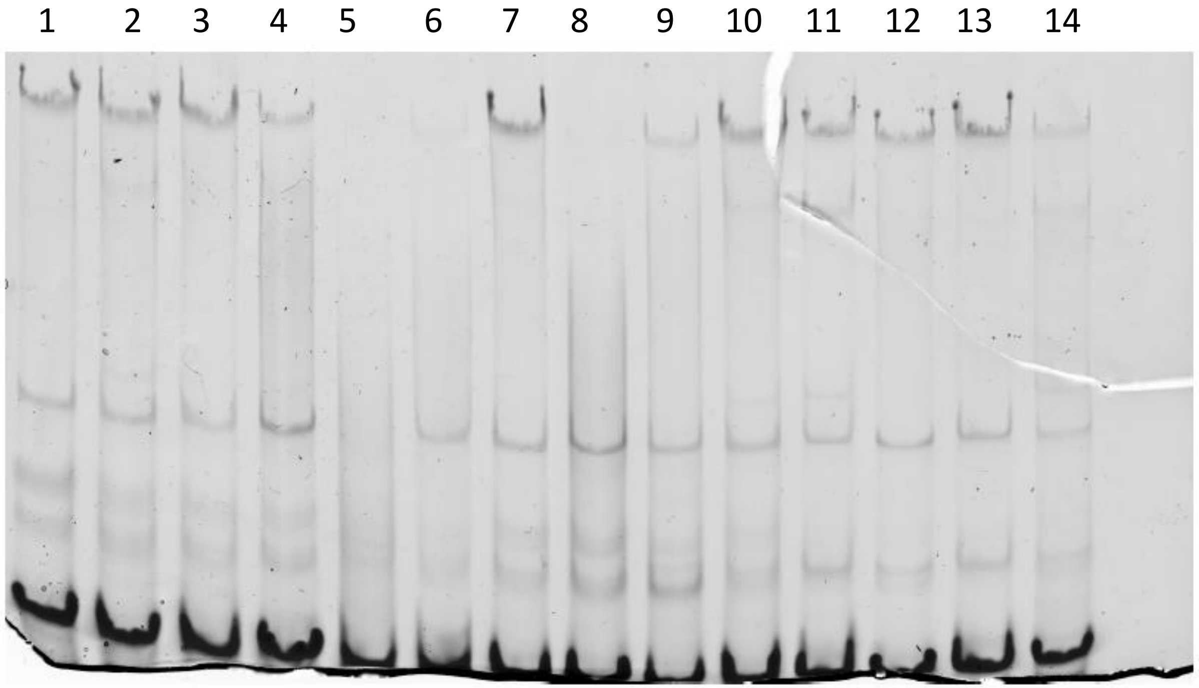

1: Control
2: Control
3: Disease control
4: DI:1
5: not included
6: XIII:1
7: control
8: XII:3
9: YII:3
10: control
11-14: not included
