# Supplementary figures and images for: Digenic inheritance involving a muscle-specific protein kinase and the giant titin protein causes a skeletal muscle myopathy
Source: Nat Genet. 2024 Mar 1;56(3):395–407. doi: 10.1038/s41588-023-01651-0 (PMC10937387; doi:10.1038/s41588-023-01651-0)

U N P

U      N      P

U      N      P

U      N      P

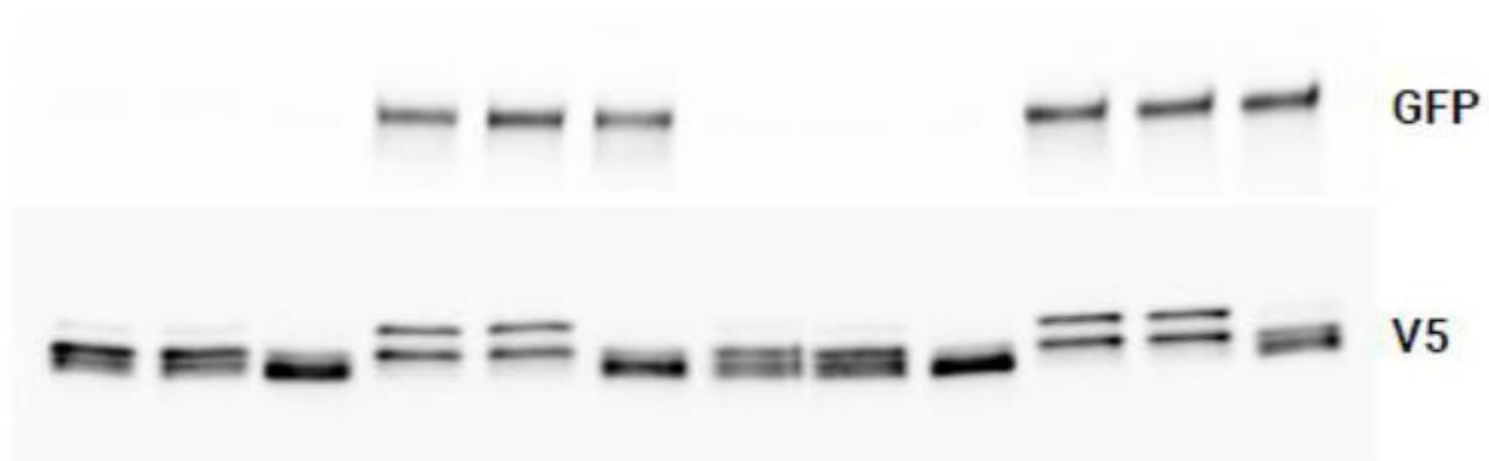

Supplement: Supplementary file 8 — Unprocessed western blots. [file 41588_2023_1651_MOESM8_ESM.pdf]

**a**

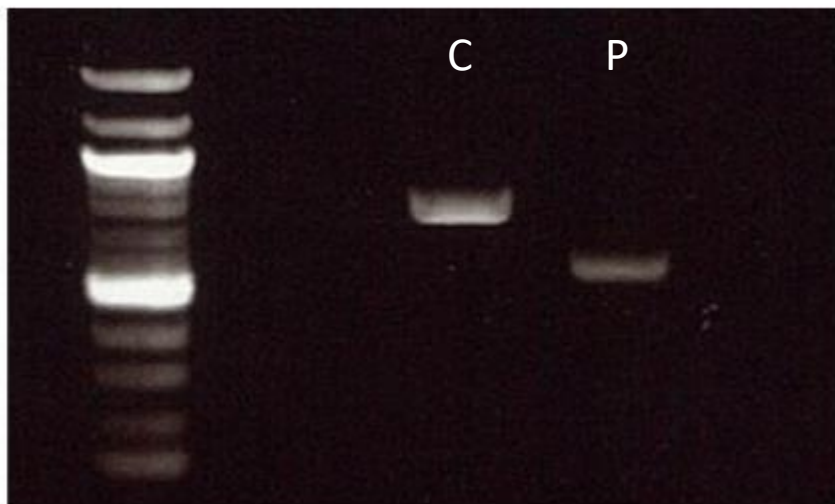

**b**

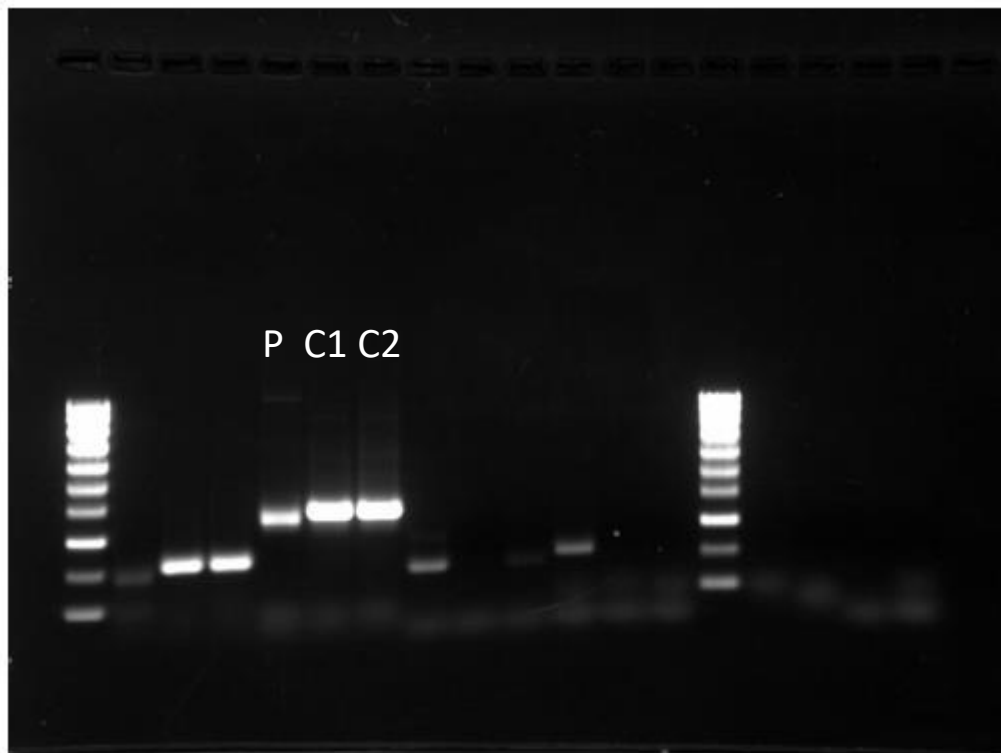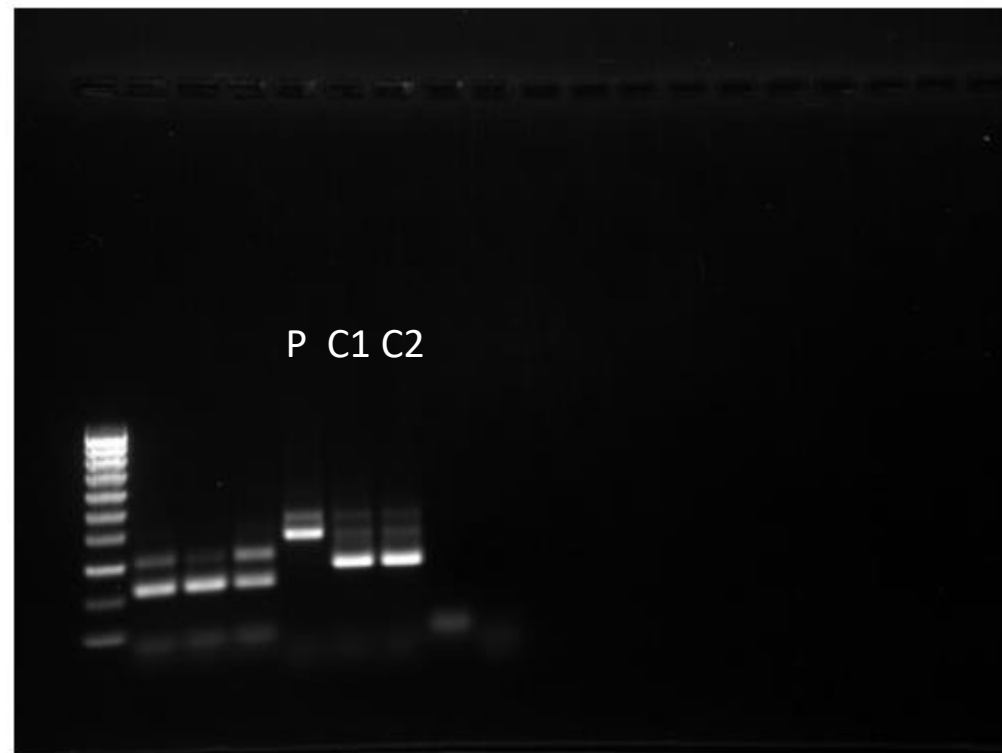

Supplement: Supplementary file 9 — Unprocessed gels. [file 41588_2023_1651_MOESM9_ESM.pdf]

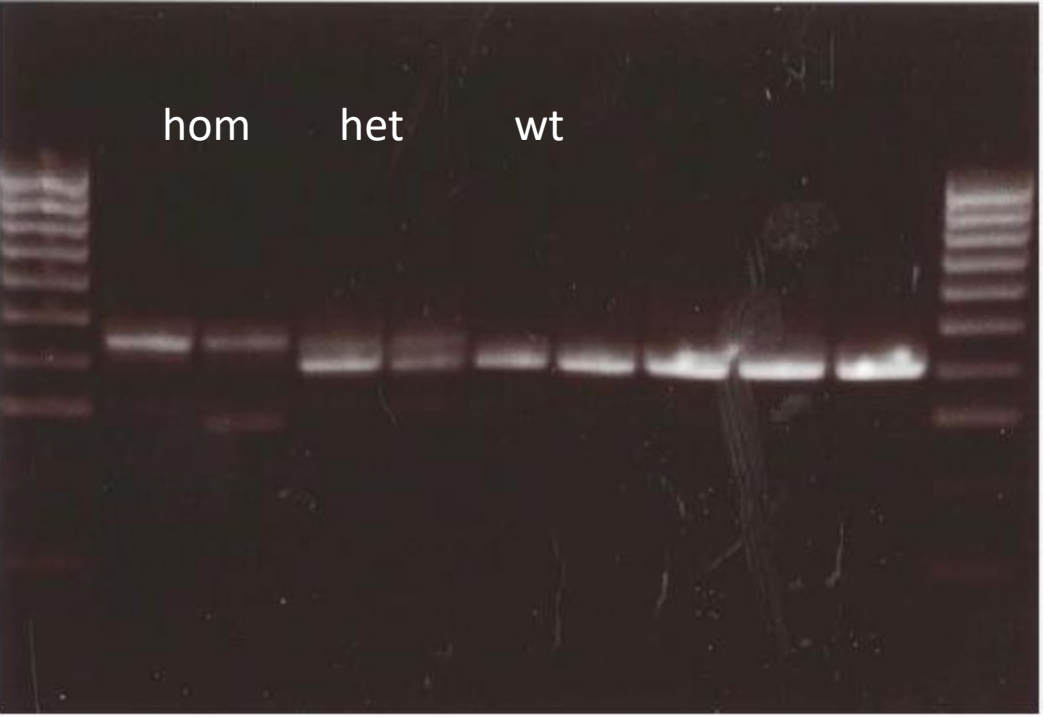

Supplement: Supplementary file 10 — Unprocessed gels. [file 41588_2023_1651_MOESM10_ESM.pdf]
